# Supplementary figures and images for: Paradoxical imbalance between activated lymphocyte protein synthesis capacity and rapid division rate
Source: eLife. 2024 Mar 21;12:RP89015. doi: 10.7554/eLife.89015 (PMC10957176; doi:10.7554/eLife.89015)

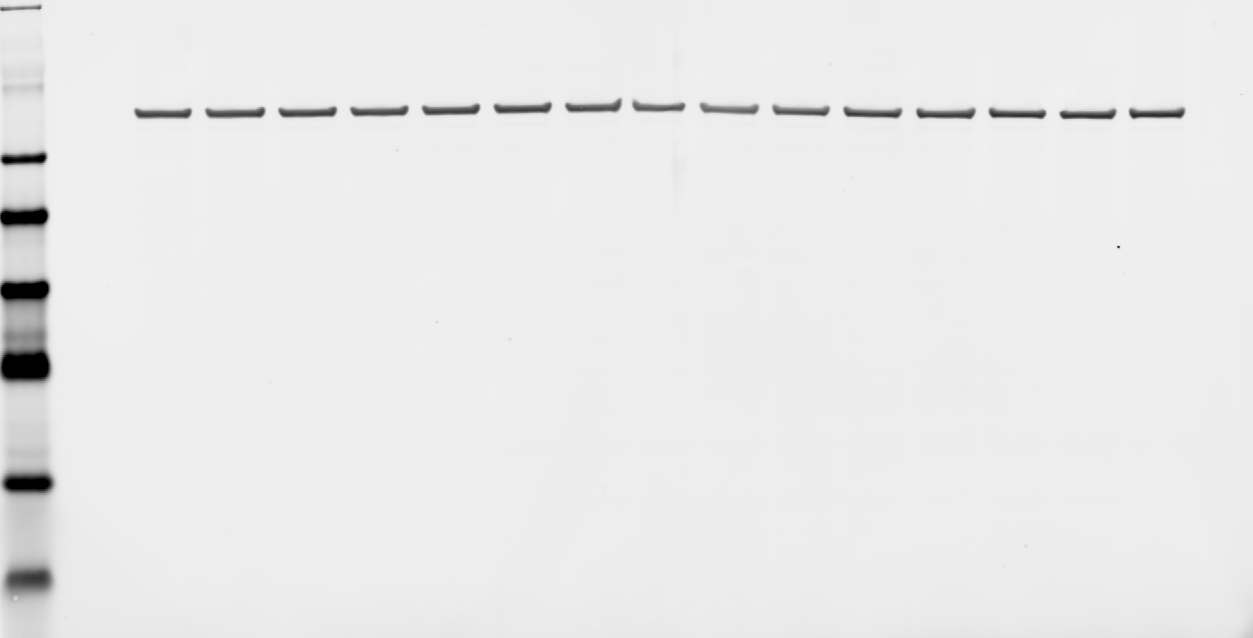

Supplement: Figure 2—source data 2. [file elife-89015-fig2-data2.zip › Figure 2C source data/Figure 2C anti-HSP90 raw blot.tif]

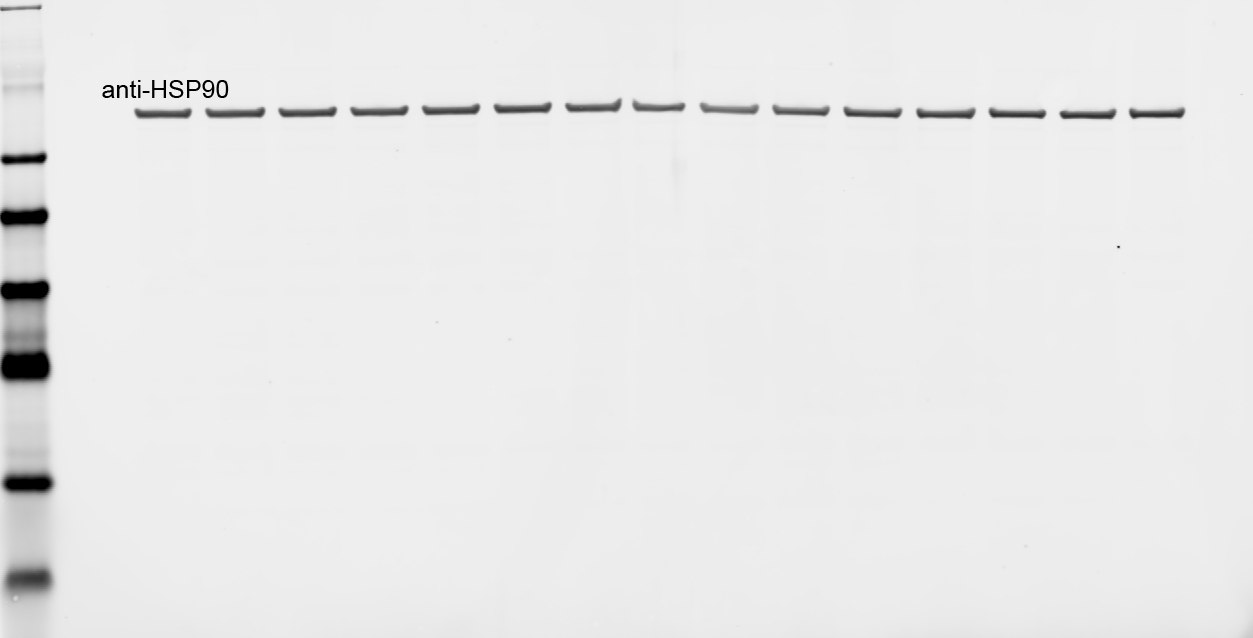

Supplement: Figure 2—source data 2. [file elife-89015-fig2-data2.zip › Figure 2C source data/Figure 2C anti-HSP90.tif]

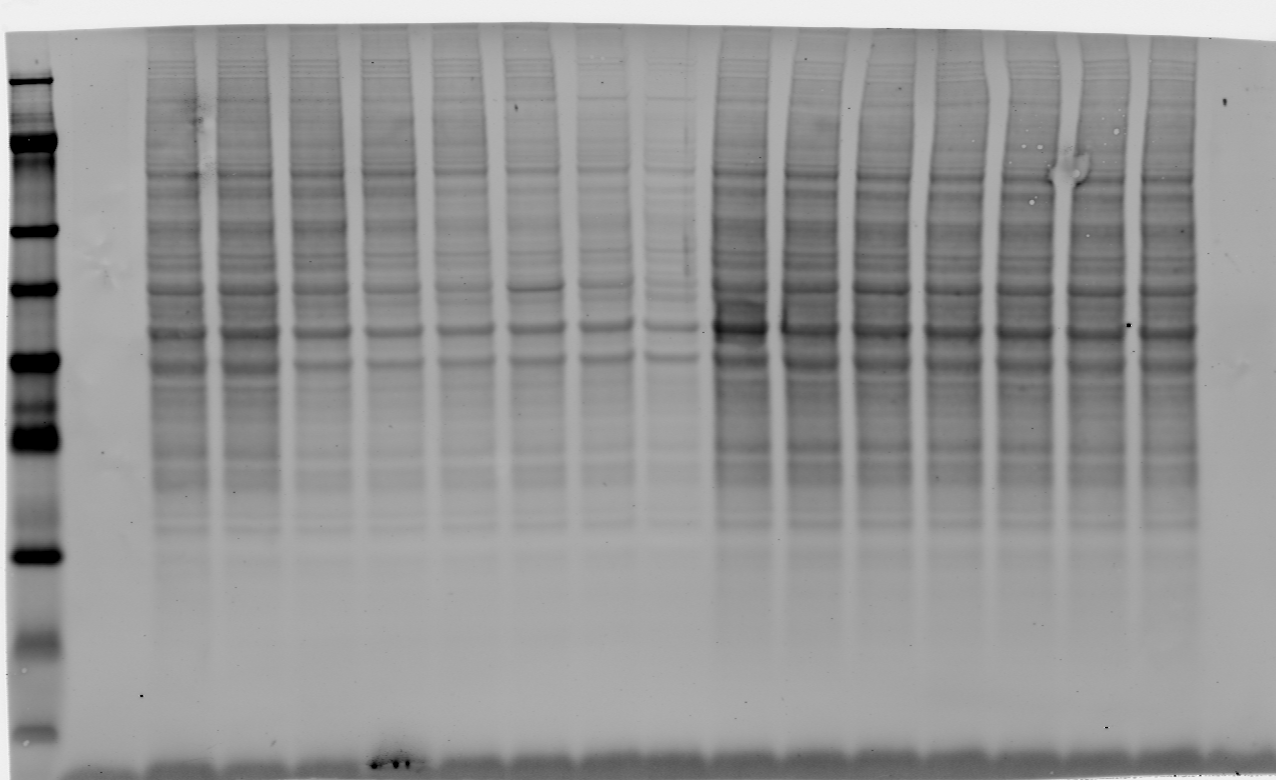

Supplement: Figure 2—source data 2. [file elife-89015-fig2-data2.zip › Figure 2C source data/Figure 2C anti-puromycin raw blot.tif]

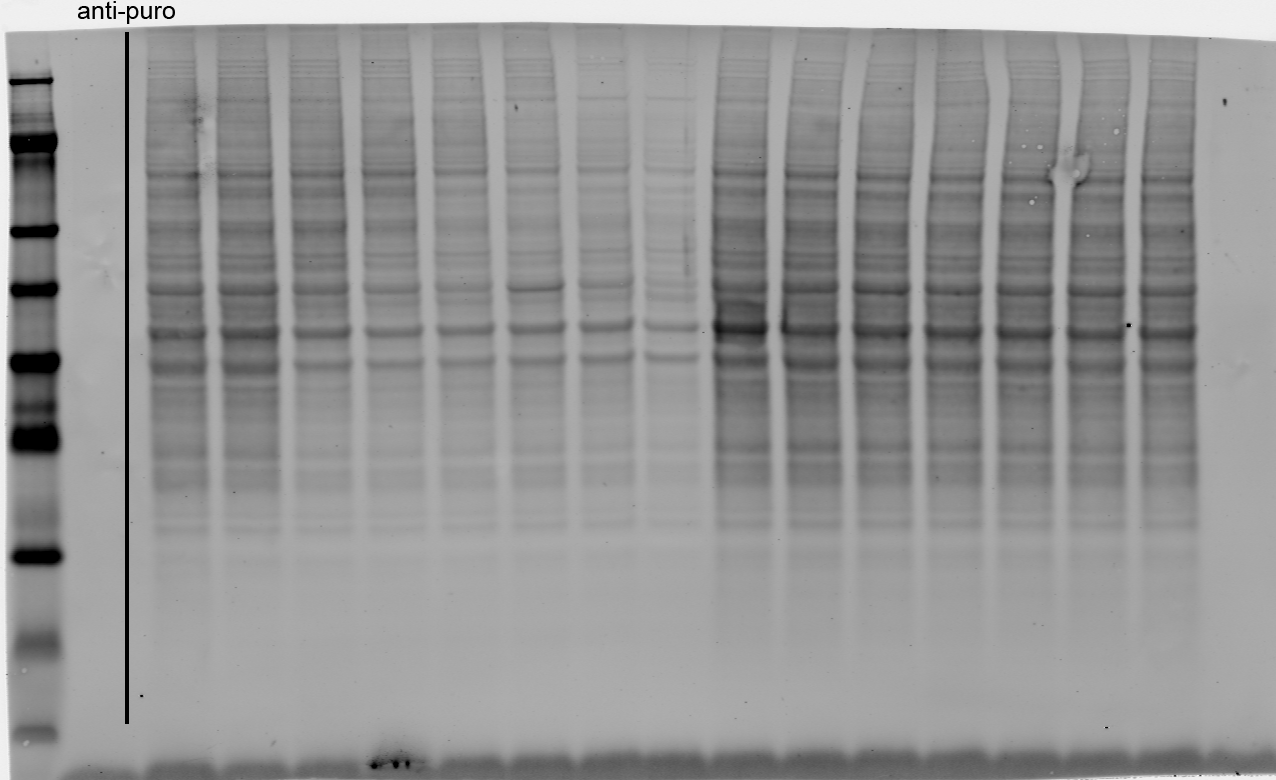

Supplement: Figure 2—source data 2. [file elife-89015-fig2-data2.zip › Figure 2C source data/Figure 2C anti-puromycin.tif]

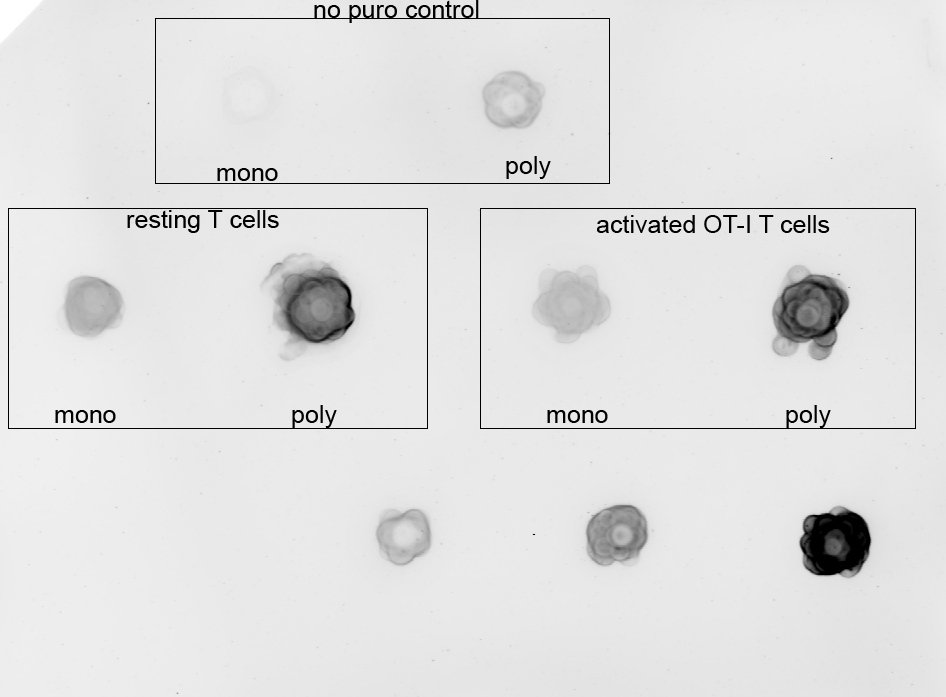

Supplement: Figure 5—source data 2. [file elife-89015-fig5-data2.zip › Figure 5A source data/Figure 5A dot blot anti-puro.tif]

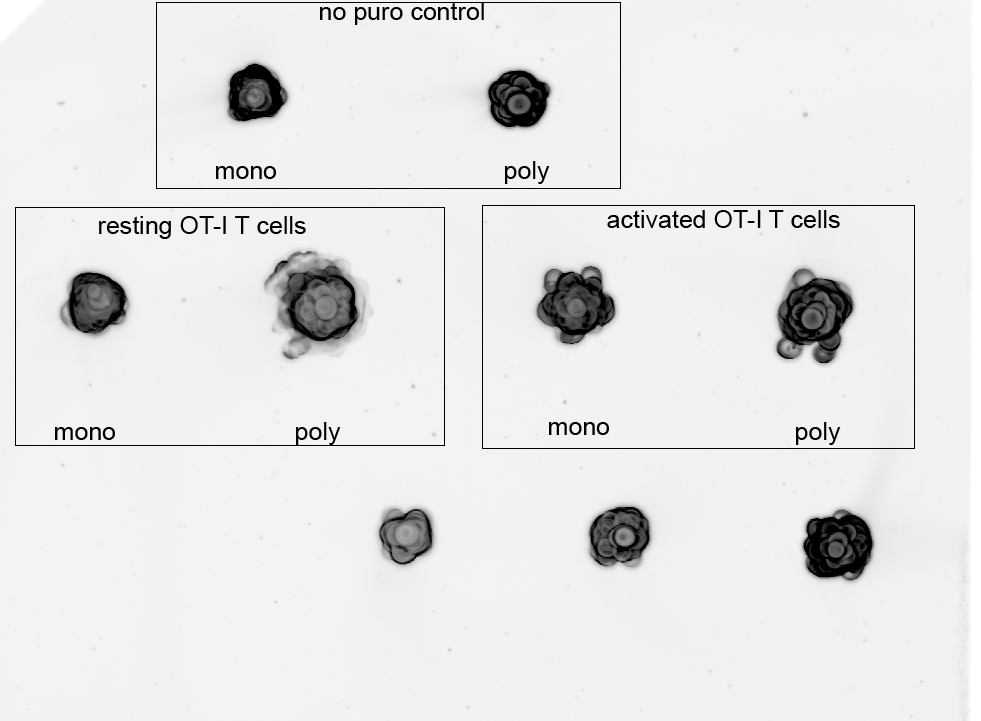

Supplement: Figure 5—source data 2. [file elife-89015-fig5-data2.zip › Figure 5A source data/Figure 5A dot blot anti-RPL7.tif]

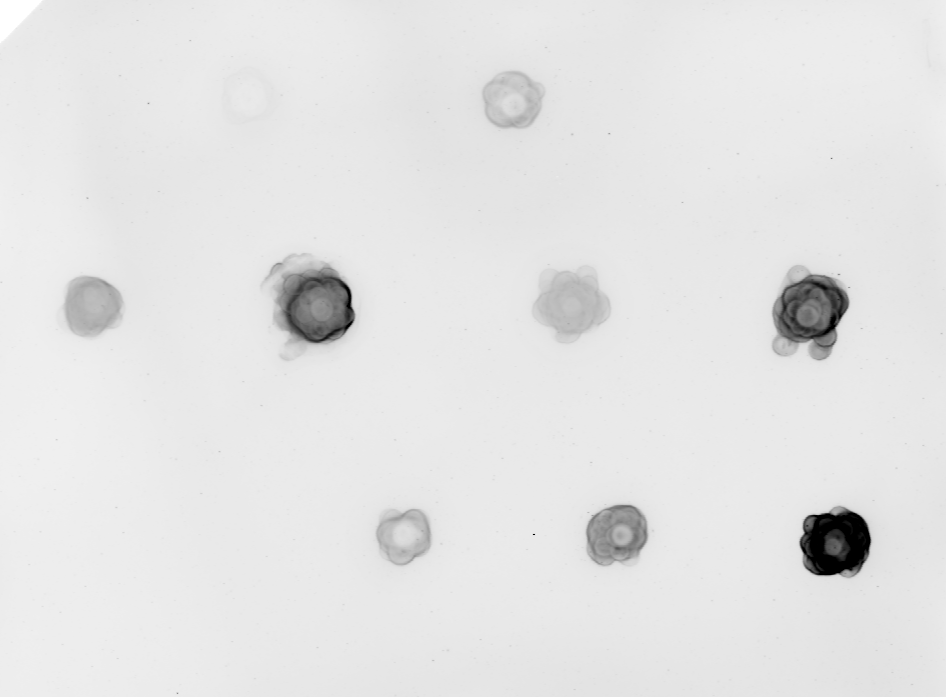

Supplement: Figure 5—source data 2. [file elife-89015-fig5-data2.zip › Figure 5A source data/Figure 5A raw dot blot anti-puro.tif]

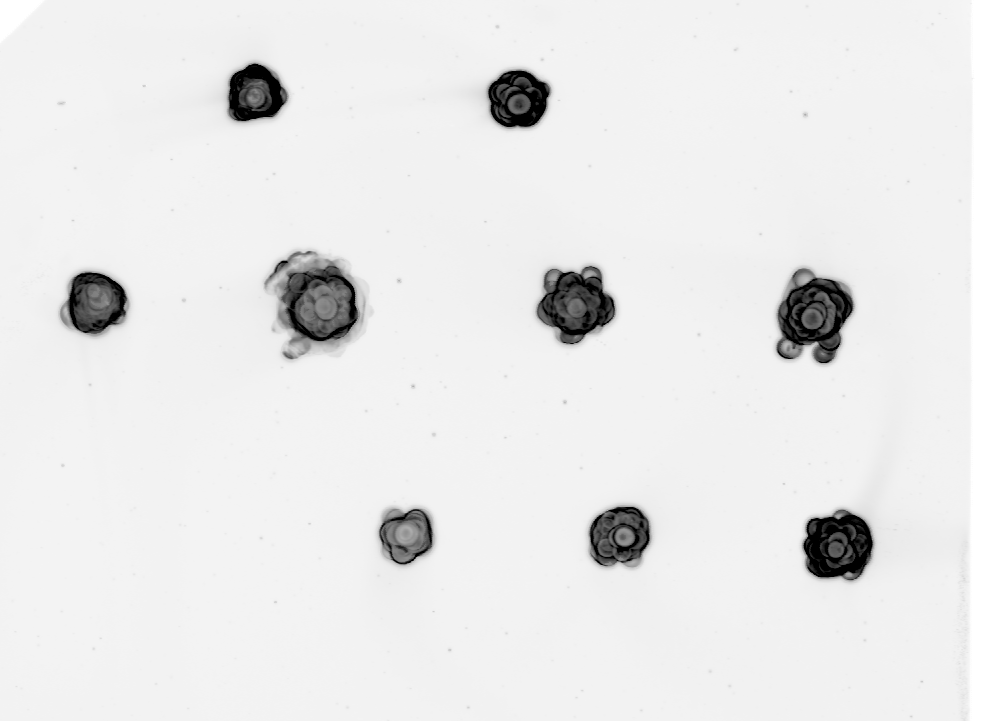

Supplement: Figure 5—source data 2. [file elife-89015-fig5-data2.zip › Figure 5A source data/Figure 5A raw dot blot anti-RPL7.tif]

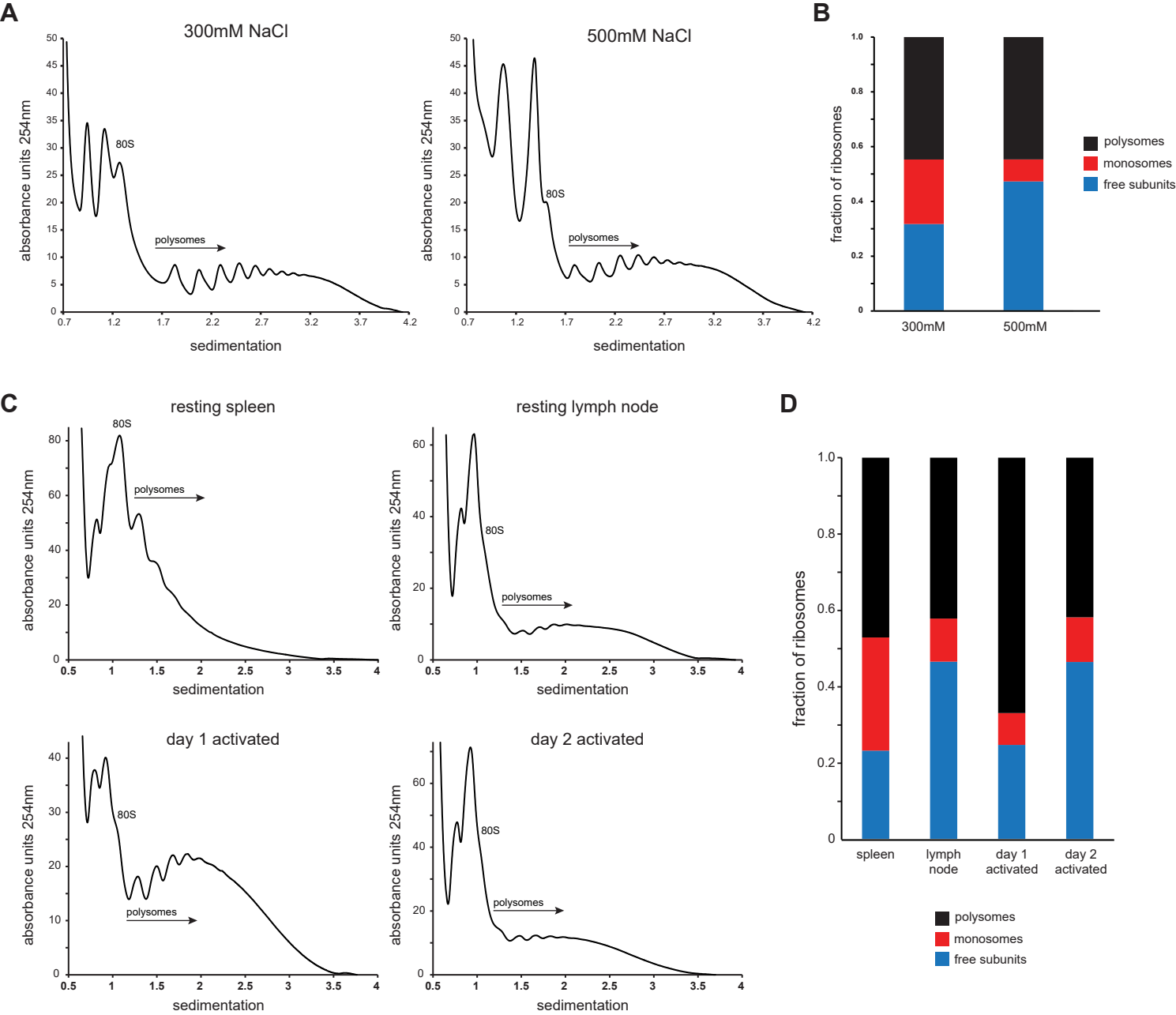

Supplement: Figure 5—figure supplement 2—source data 1. [file elife-89015-fig5-figsupp2-data1.pdf]

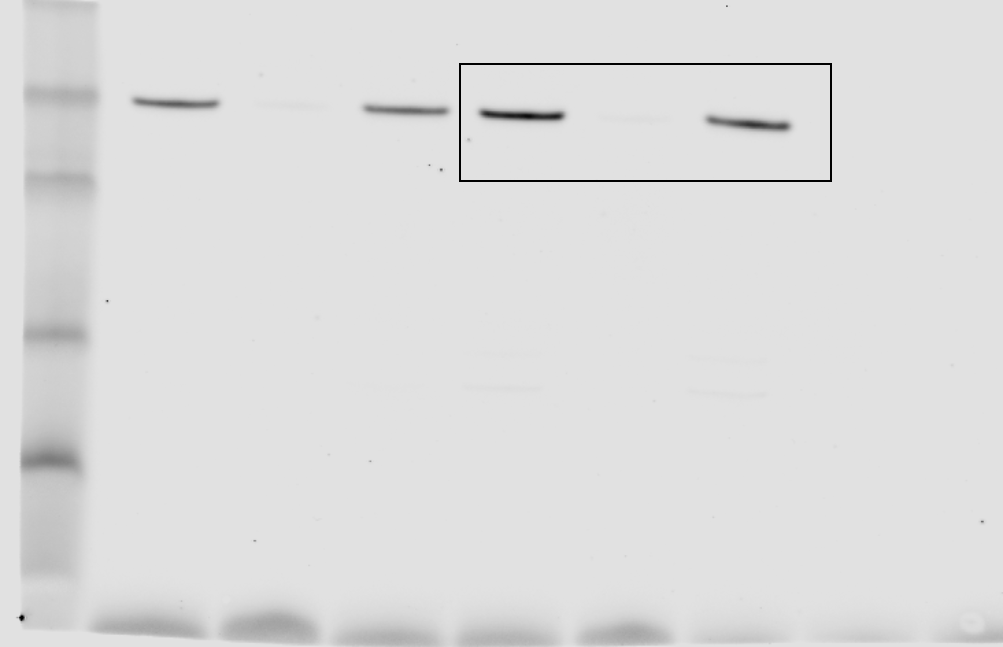

Supplement: Figure 6—figure supplement 1—source data 1. [file elife-89015-fig6-figsupp1-data1.zip › Figure 6-figure supplement 1 blots/A - HeLa fibrillarin.tif]

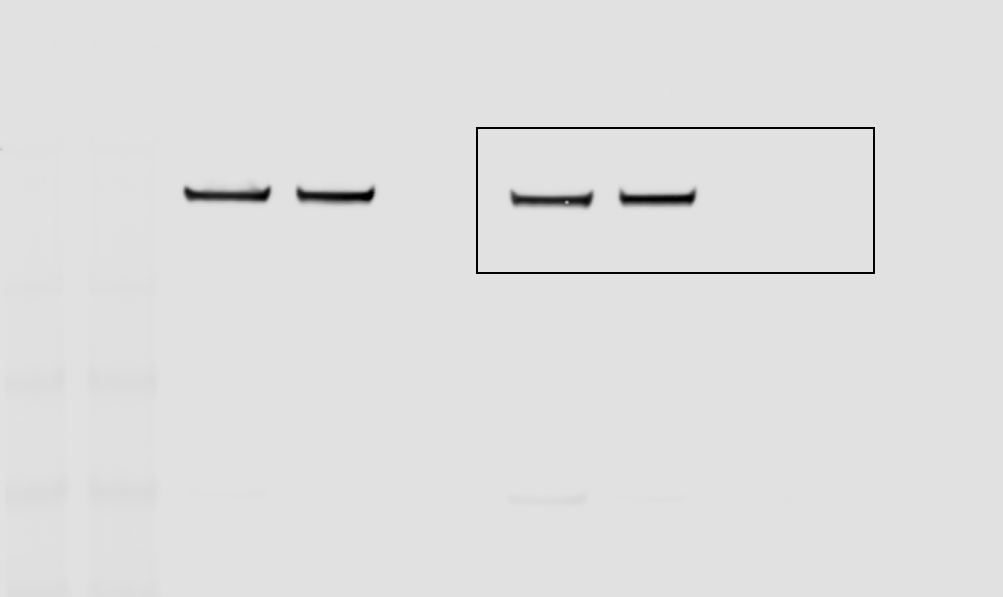

Supplement: Figure 6—figure supplement 1—source data 1. [file elife-89015-fig6-figsupp1-data1.zip › Figure 6-figure supplement 1 blots/A - HeLa GRP94.tif]

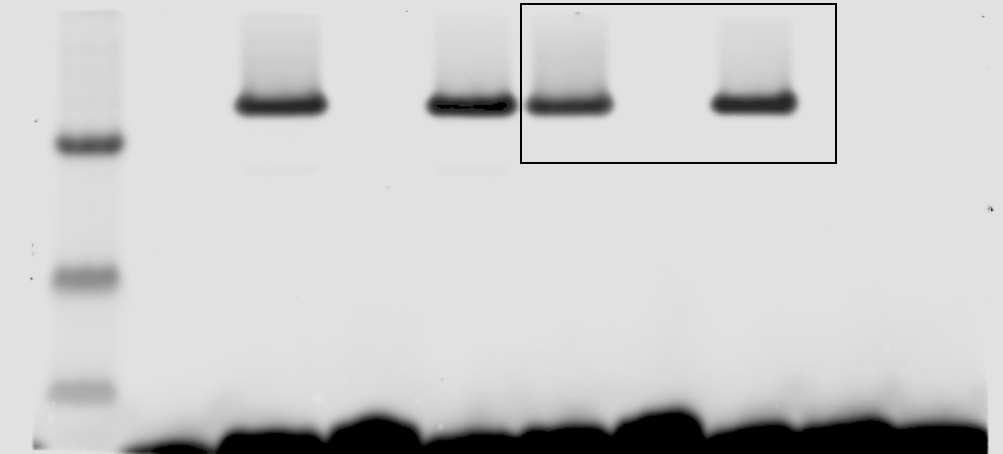

Supplement: Figure 6—figure supplement 1—source data 1. [file elife-89015-fig6-figsupp1-data1.zip › Figure 6-figure supplement 1 blots/A - HeLa histone H3.tif]

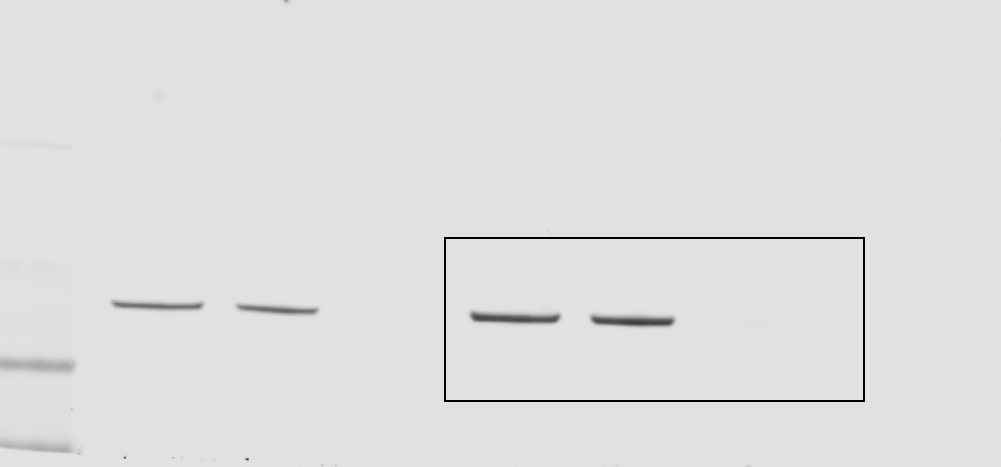

Supplement: Figure 6—figure supplement 1—source data 1. [file elife-89015-fig6-figsupp1-data1.zip › Figure 6-figure supplement 1 blots/A - HeLa HSP90.tif]

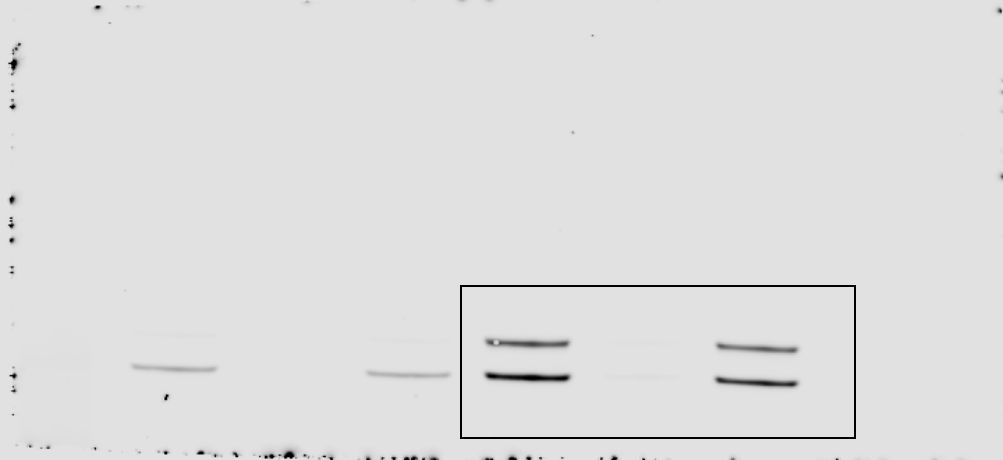

Supplement: Figure 6—figure supplement 1—source data 1. [file elife-89015-fig6-figsupp1-data1.zip › Figure 6-figure supplement 1 blots/A - HeLa lamin.tif]

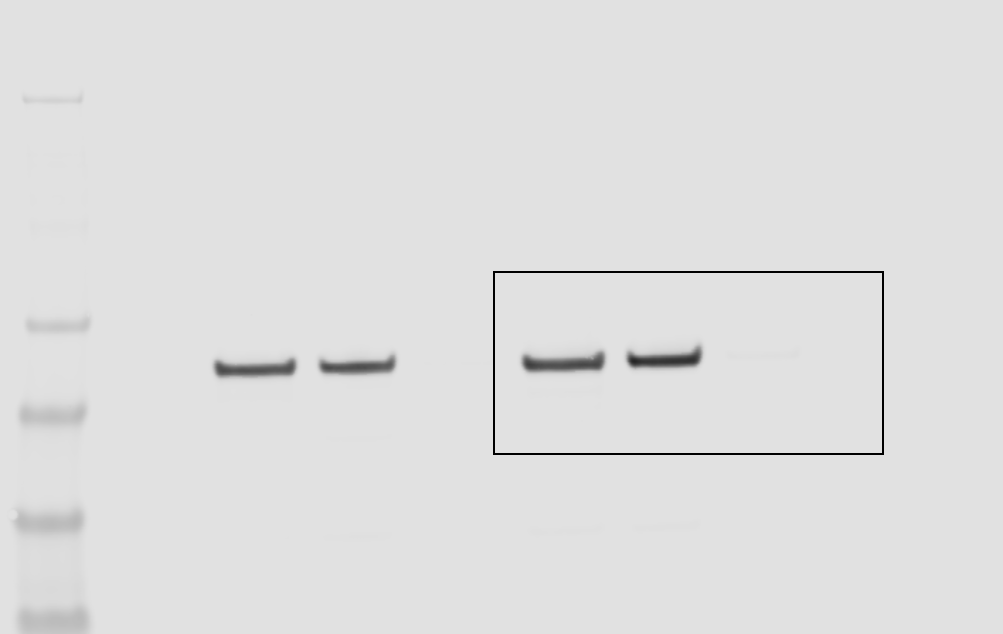

Supplement: Figure 6—figure supplement 1—source data 1. [file elife-89015-fig6-figsupp1-data1.zip › Figure 6-figure supplement 1 blots/A - HeLa PDI.tif]

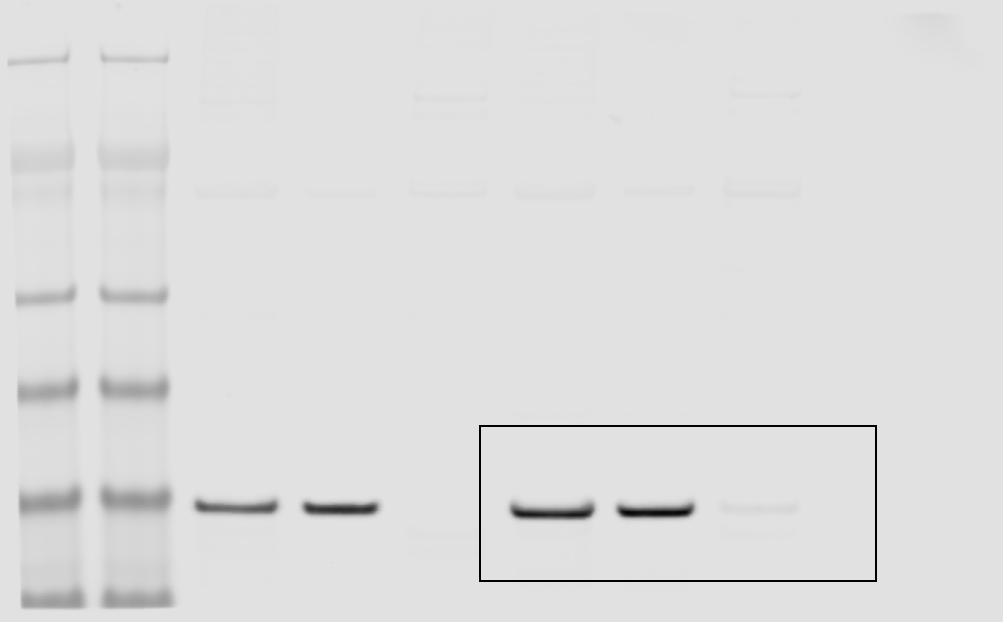

Supplement: Figure 6—figure supplement 1—source data 1. [file elife-89015-fig6-figsupp1-data1.zip › Figure 6-figure supplement 1 blots/A - HeLa riboP.tif]

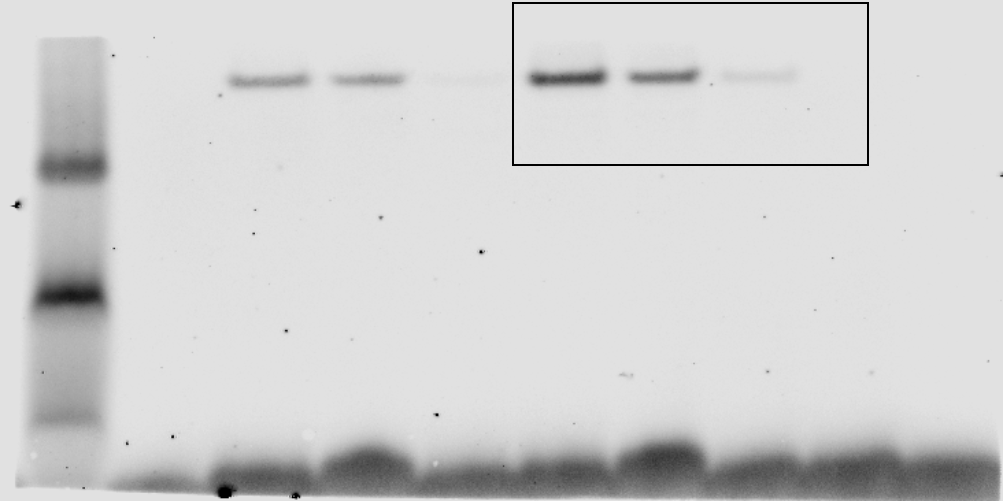

Supplement: Figure 6—figure supplement 1—source data 1. [file elife-89015-fig6-figsupp1-data1.zip › Figure 6-figure supplement 1 blots/A - HeLa RPL26.tif]

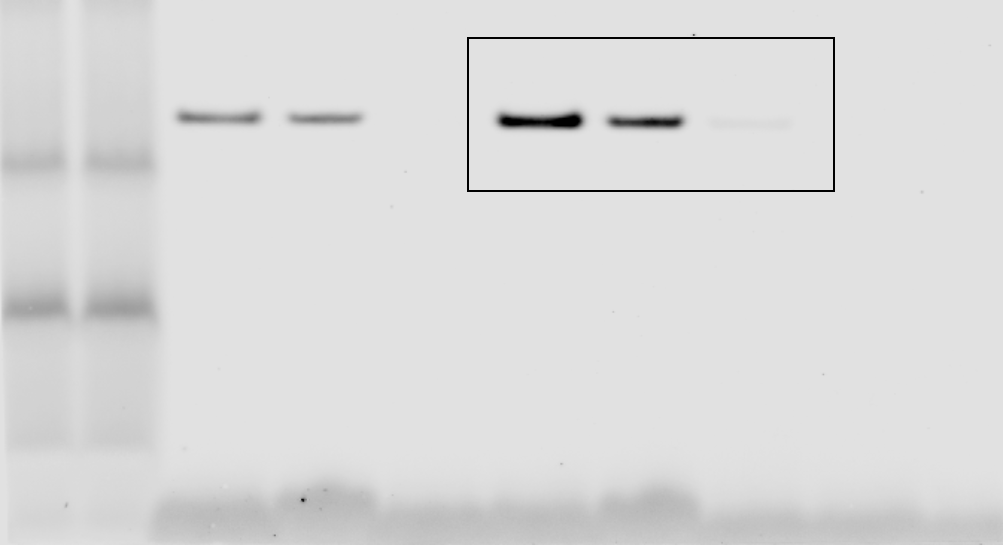

Supplement: Figure 6—figure supplement 1—source data 1. [file elife-89015-fig6-figsupp1-data1.zip › Figure 6-figure supplement 1 blots/A - HeLa RPL28.tif]

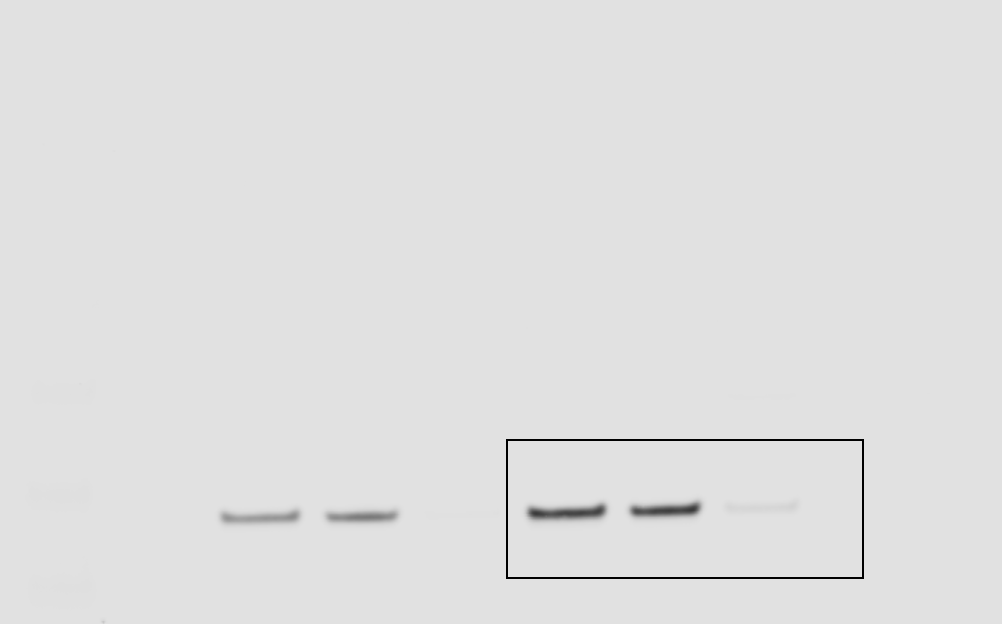

Supplement: Figure 6—figure supplement 1—source data 1. [file elife-89015-fig6-figsupp1-data1.zip › Figure 6-figure supplement 1 blots/A - HeLa RPL5.tif]

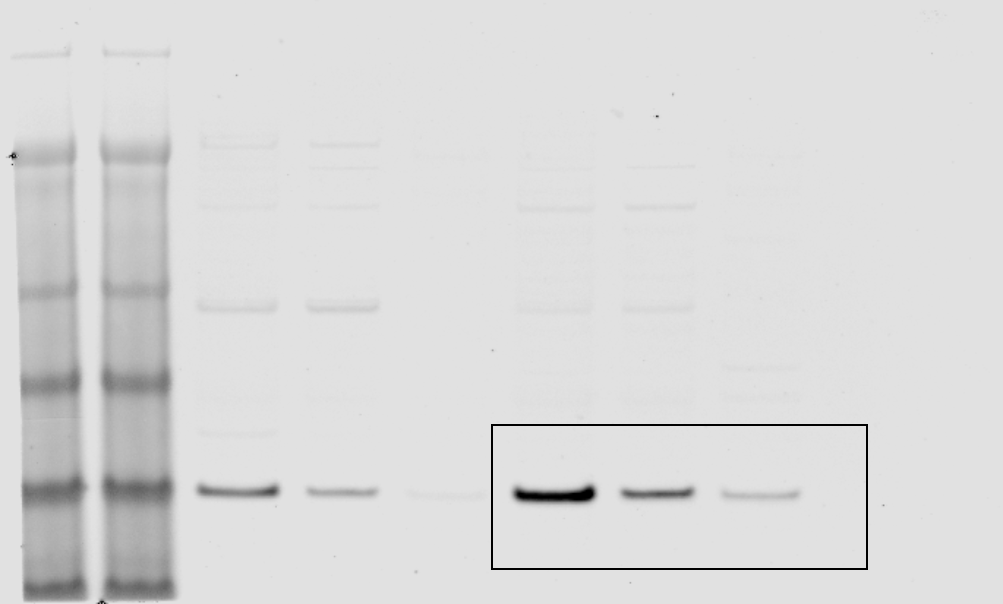

Supplement: Figure 6—figure supplement 1—source data 1. [file elife-89015-fig6-figsupp1-data1.zip › Figure 6-figure supplement 1 blots/A - HeLa RPL6.tif]

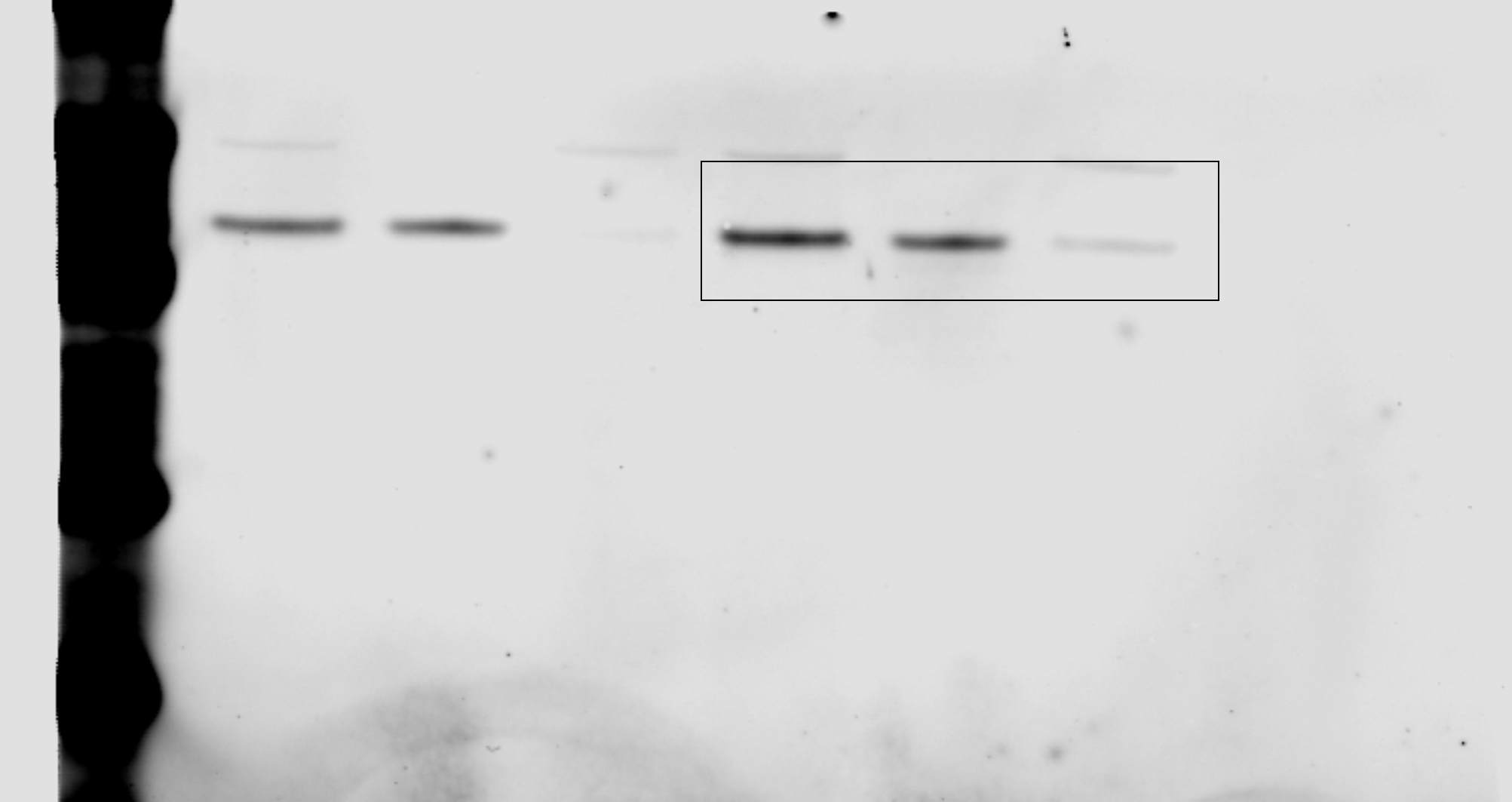

Supplement: Figure 6—figure supplement 1—source data 1. [file elife-89015-fig6-figsupp1-data1.zip › Figure 6-figure supplement 1 blots/A - HeLa RPS6.tif]

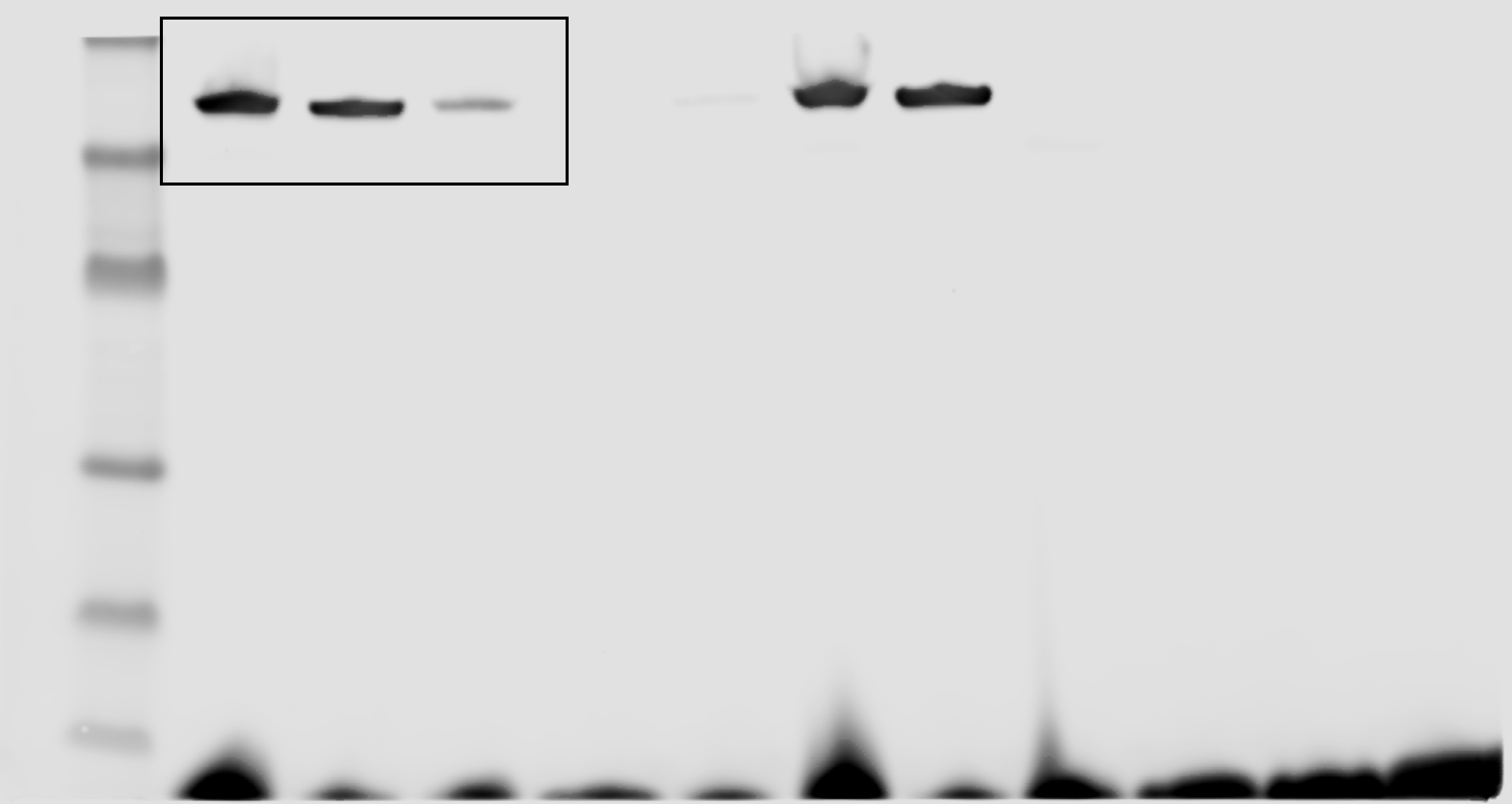

Supplement: Figure 6—figure supplement 1—source data 1. [file elife-89015-fig6-figsupp1-data1.zip › Figure 6-figure supplement 1 blots/B - resting OT-I actin.tif]

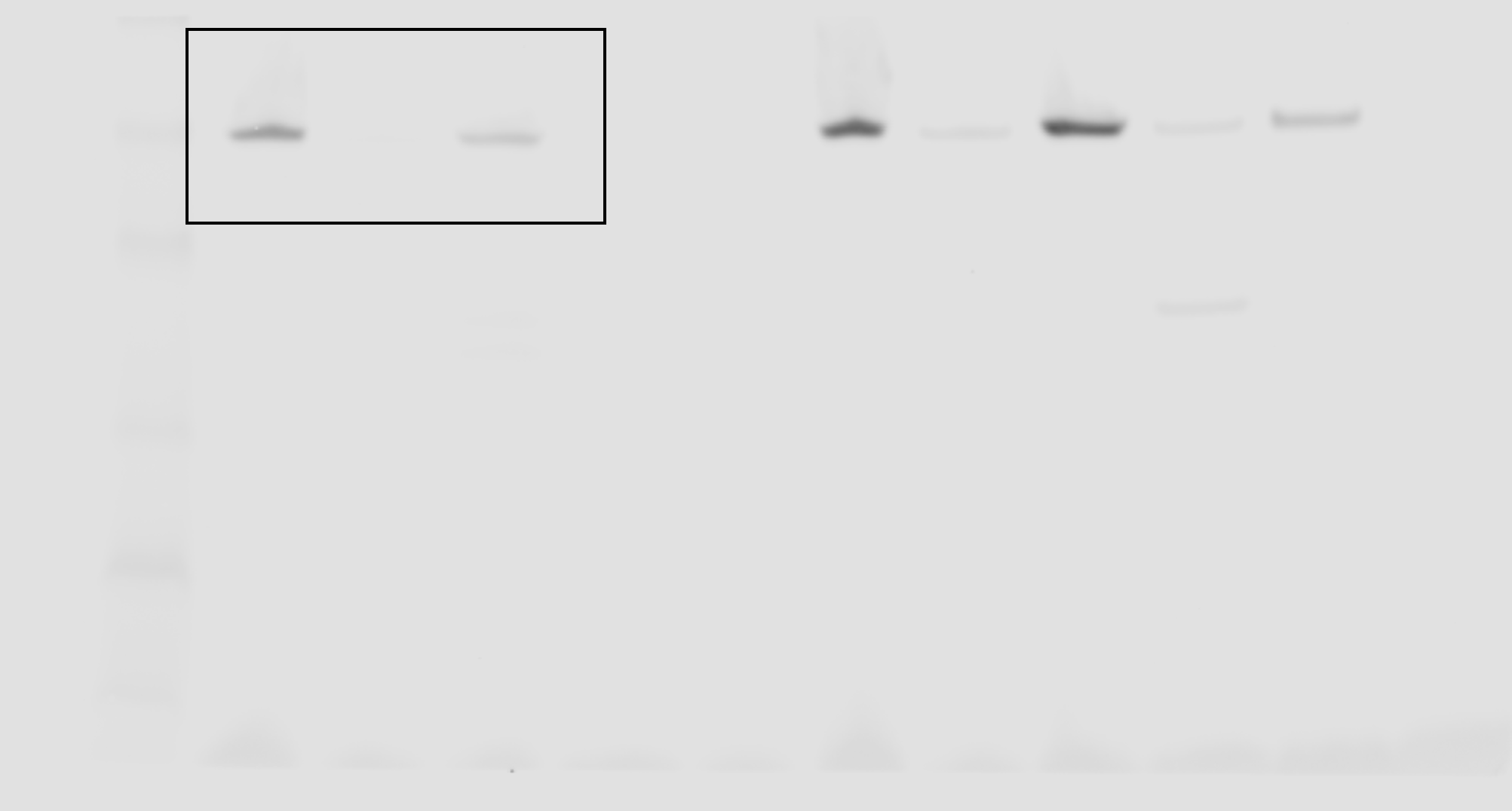

Supplement: Figure 6—figure supplement 1—source data 1. [file elife-89015-fig6-figsupp1-data1.zip › Figure 6-figure supplement 1 blots/B - resting OT-I fibrillarin.tif]

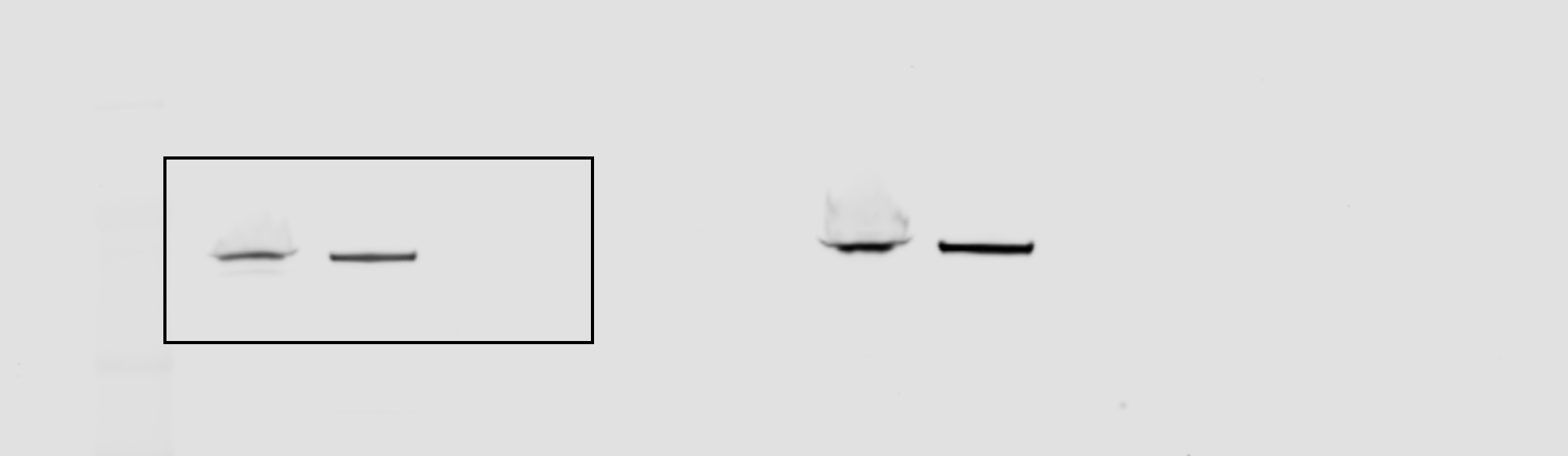

Supplement: Figure 6—figure supplement 1—source data 1. [file elife-89015-fig6-figsupp1-data1.zip › Figure 6-figure supplement 1 blots/B - resting OT-I GRP94.tif]

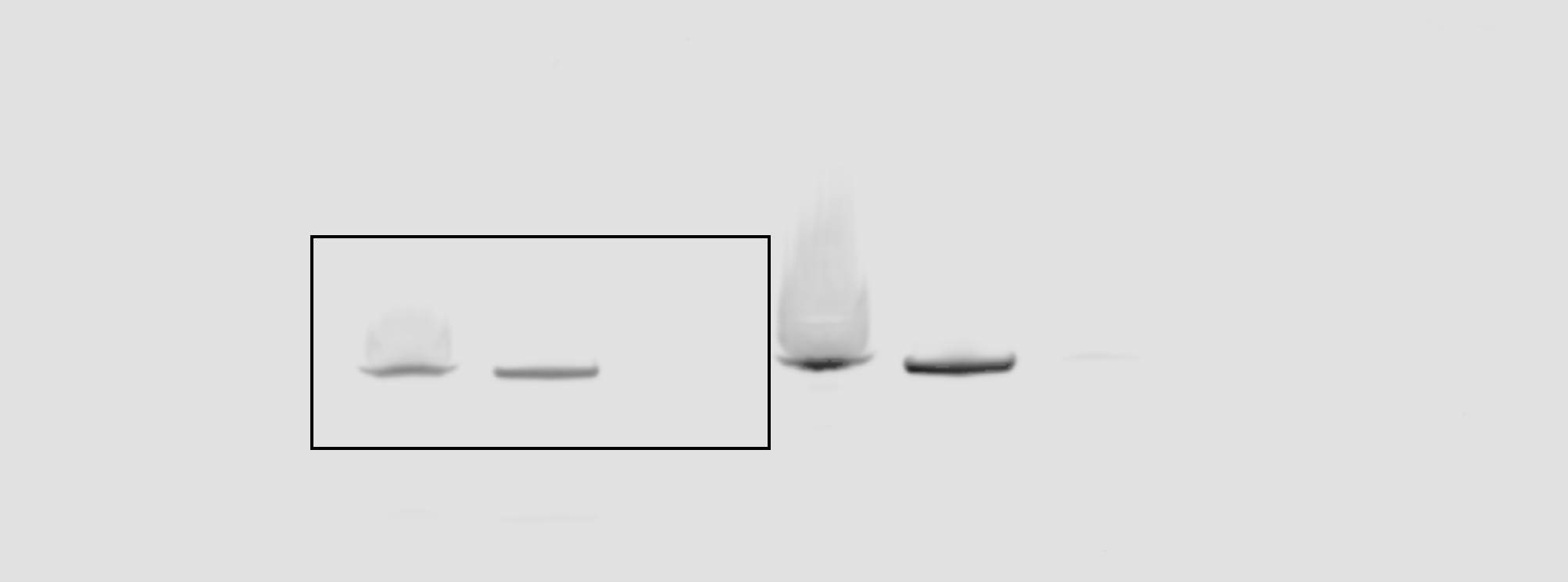

Supplement: Figure 6—figure supplement 1—source data 1. [file elife-89015-fig6-figsupp1-data1.zip › Figure 6-figure supplement 1 blots/B - resting OT-I HSP90.tif]

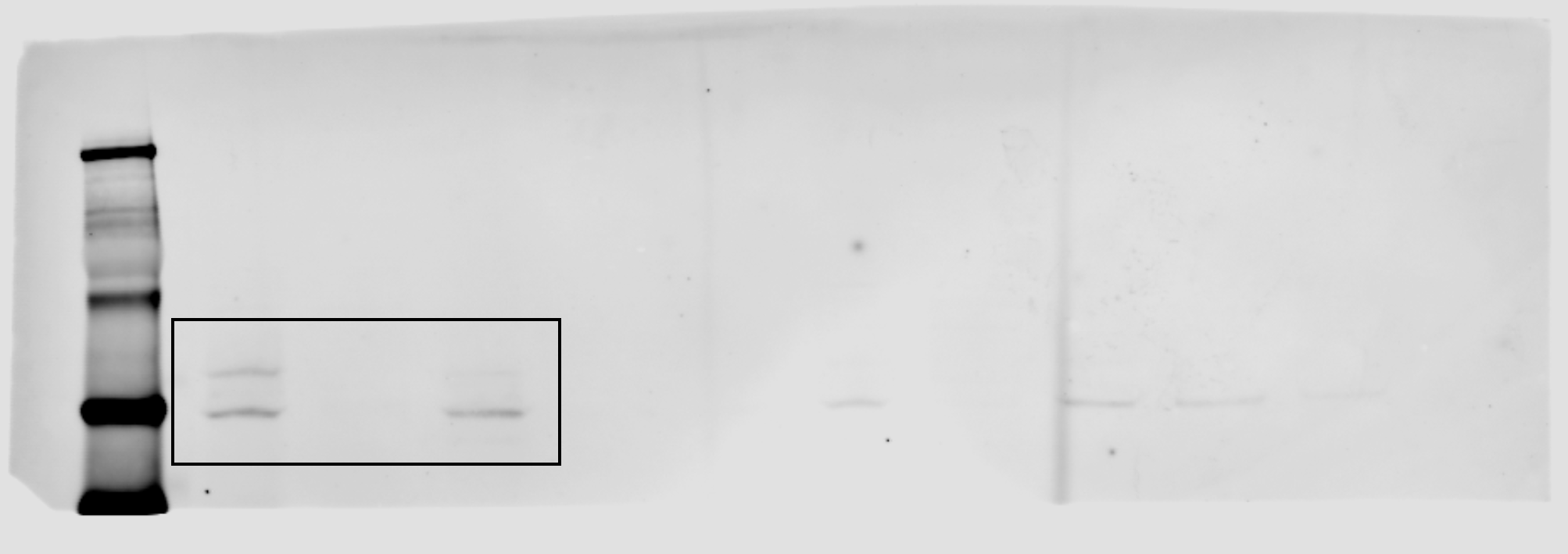

Supplement: Figure 6—figure supplement 1—source data 1. [file elife-89015-fig6-figsupp1-data1.zip › Figure 6-figure supplement 1 blots/B - resting OT-I lamin.tif]

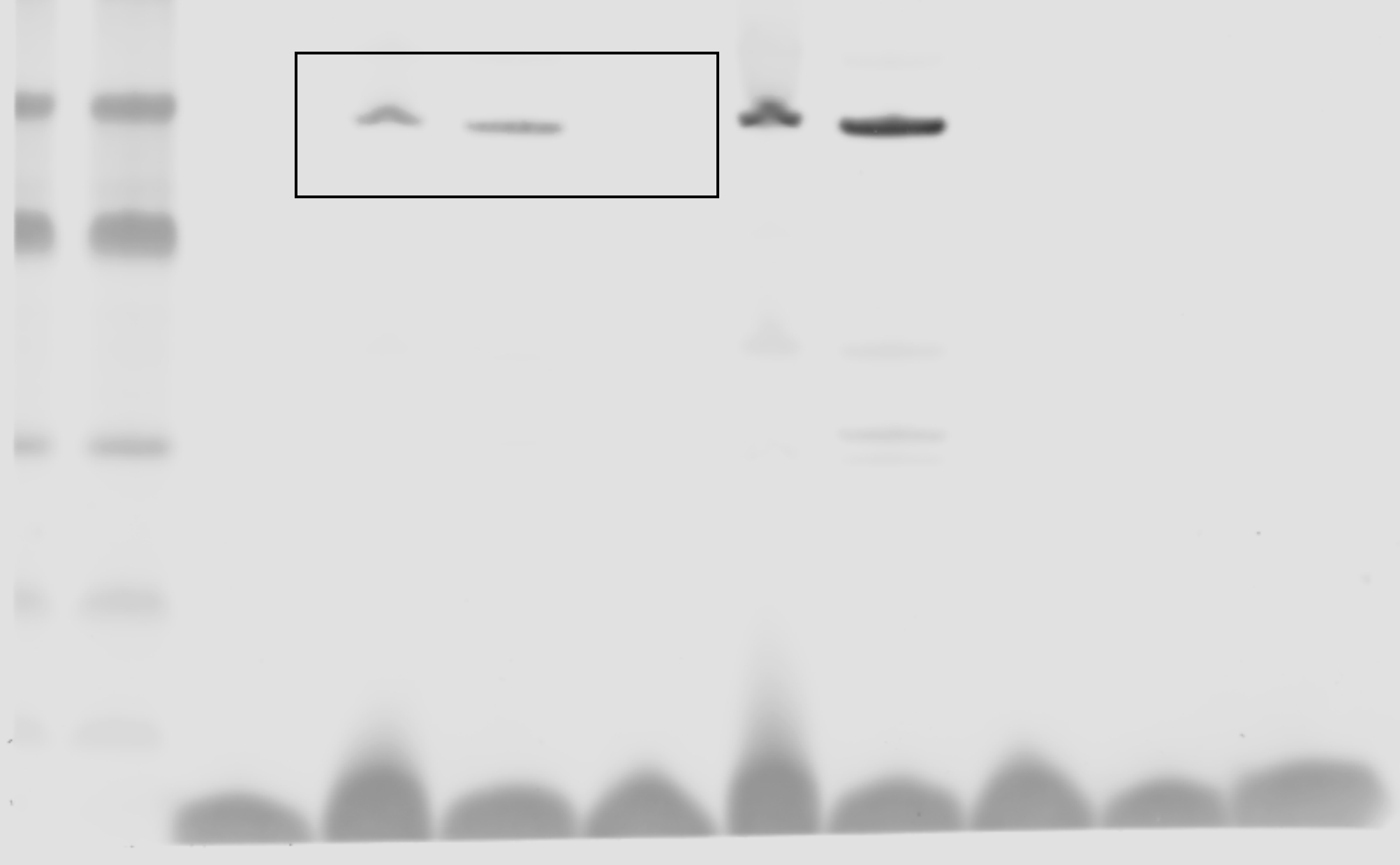

Supplement: Figure 6—figure supplement 1—source data 1. [file elife-89015-fig6-figsupp1-data1.zip › Figure 6-figure supplement 1 blots/B - resting OT-I riboP.tif]

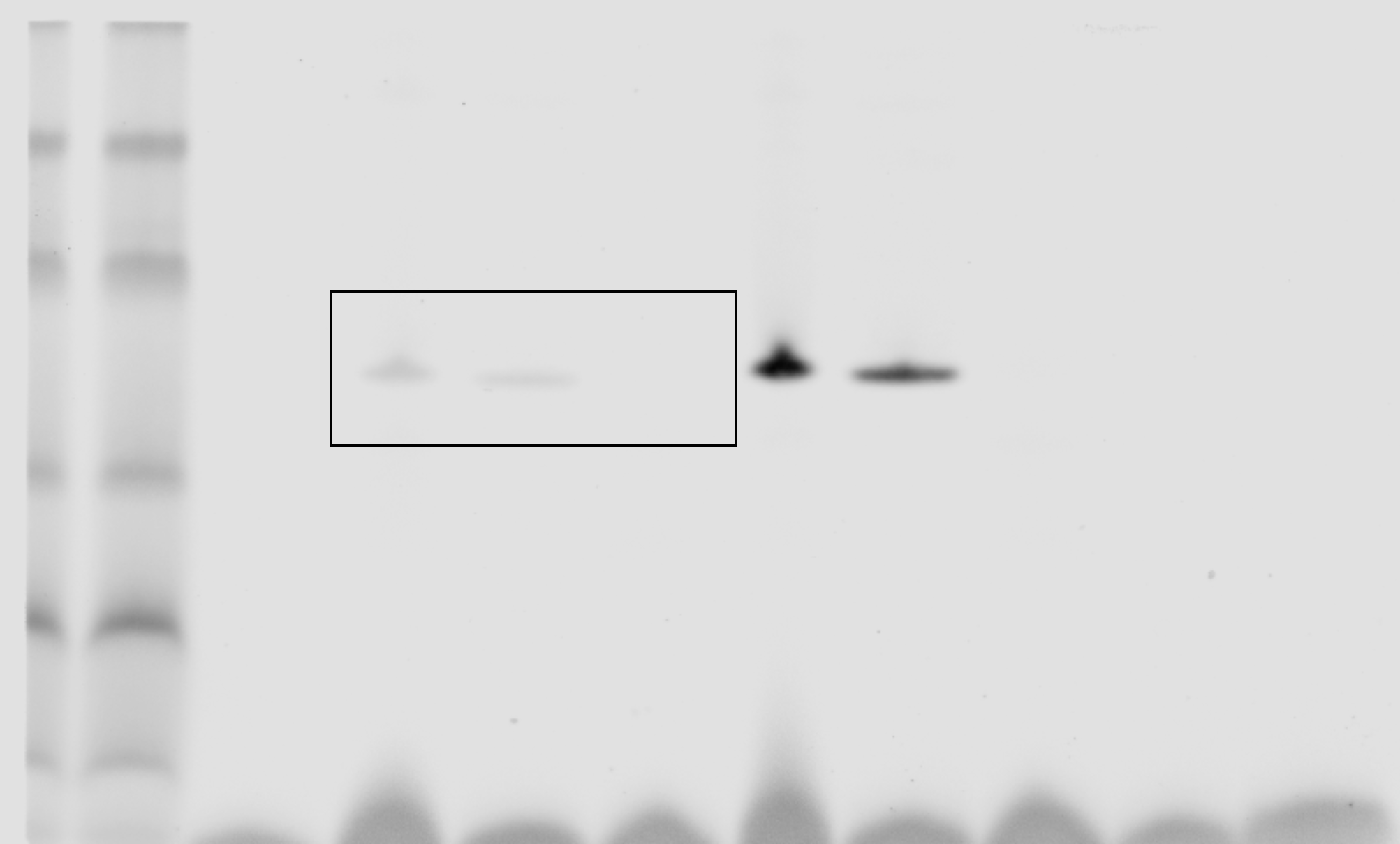

Supplement: Figure 6—figure supplement 1—source data 1. [file elife-89015-fig6-figsupp1-data1.zip › Figure 6-figure supplement 1 blots/B - resting OT-I RPL26.tif]

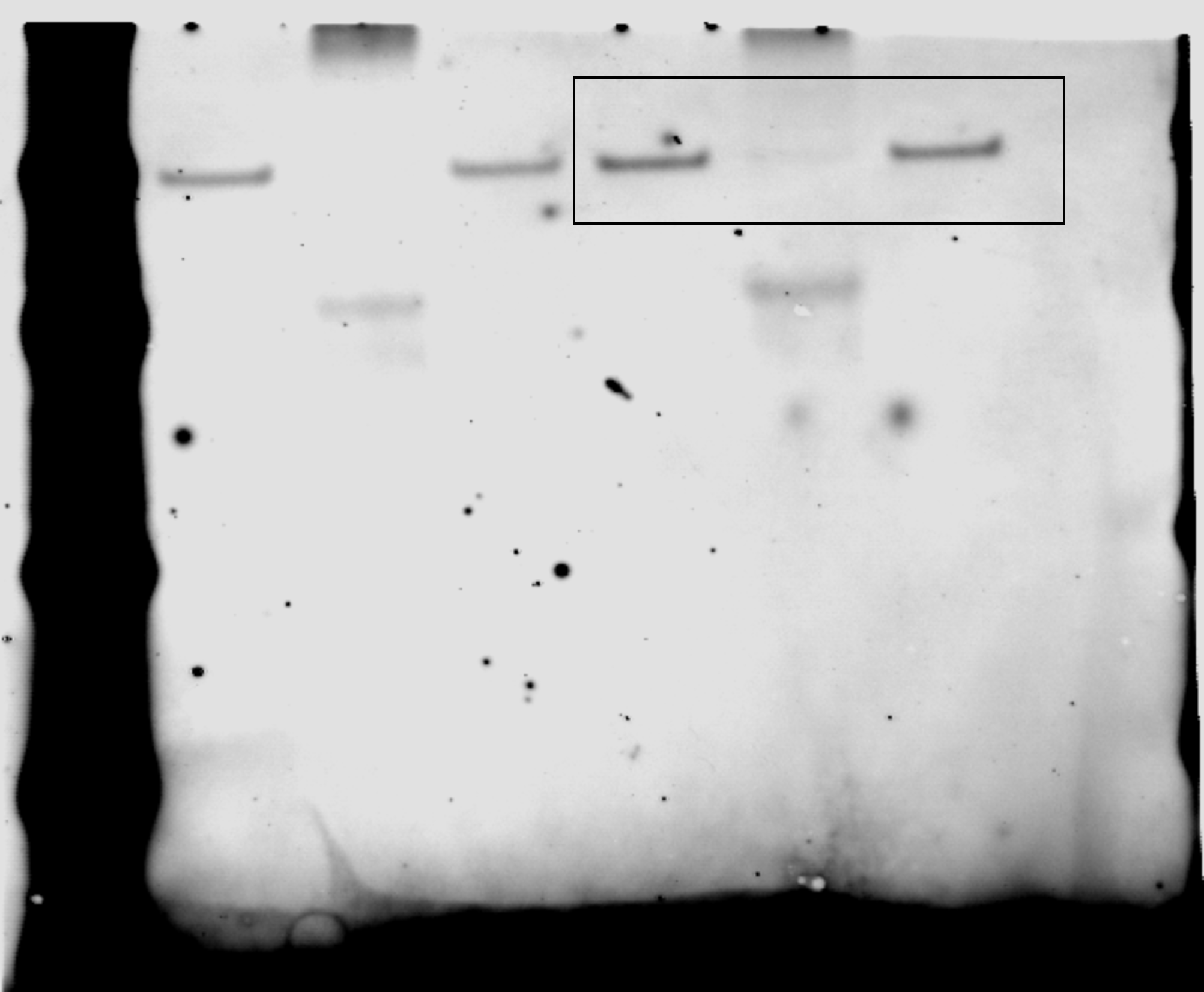

Supplement: Figure 6—figure supplement 1—source data 1. [file elife-89015-fig6-figsupp1-data1.zip › Figure 6-figure supplement 1 blots/C - act OT-I fibrillarin.tif]

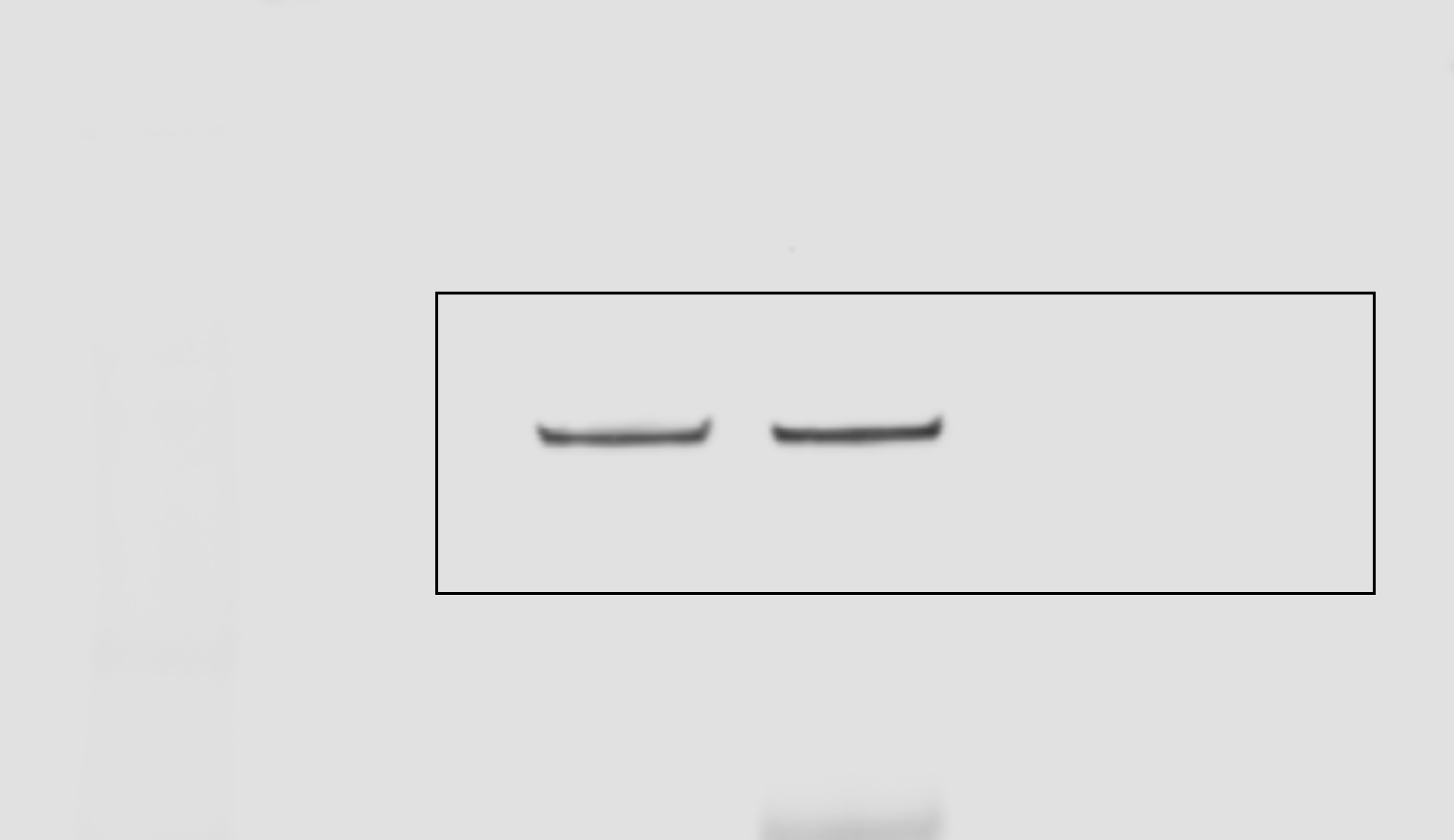

Supplement: Figure 6—figure supplement 1—source data 1. [file elife-89015-fig6-figsupp1-data1.zip › Figure 6-figure supplement 1 blots/C - act OT-I GRP94.tif]

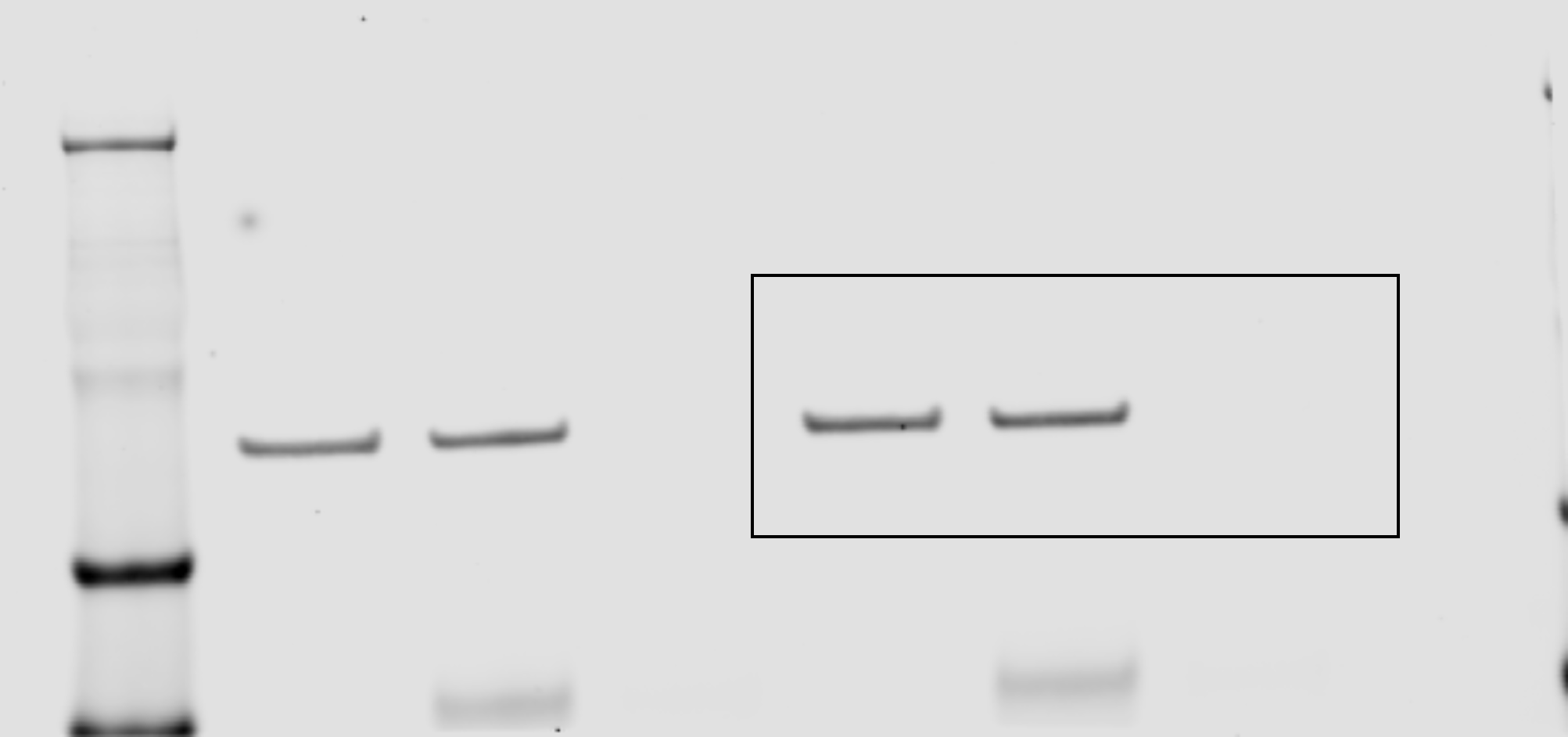

Supplement: Figure 6—figure supplement 1—source data 1. [file elife-89015-fig6-figsupp1-data1.zip › Figure 6-figure supplement 1 blots/C - act OT-I HSP90.tif]

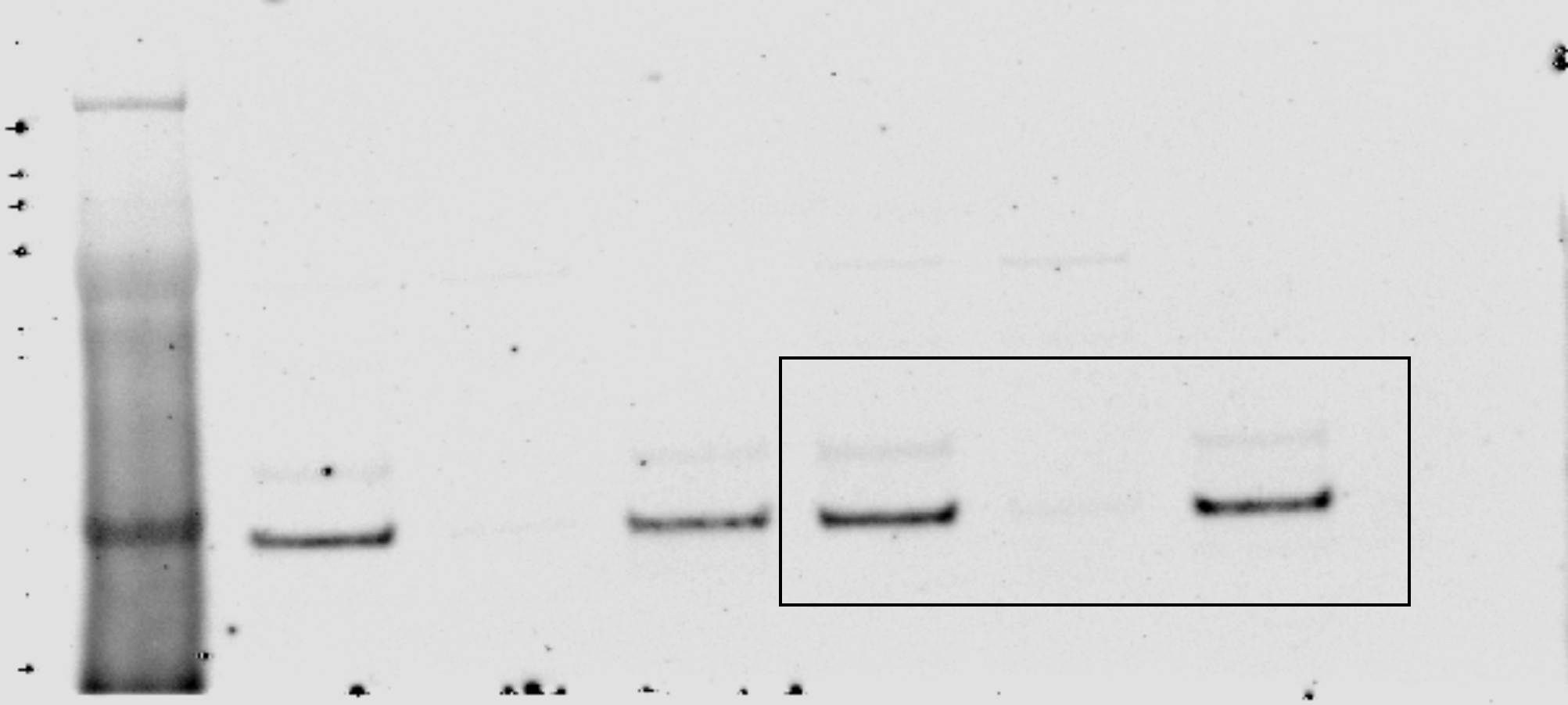

Supplement: Figure 6—figure supplement 1—source data 1. [file elife-89015-fig6-figsupp1-data1.zip › Figure 6-figure supplement 1 blots/C - act OT-I lamin.tif]

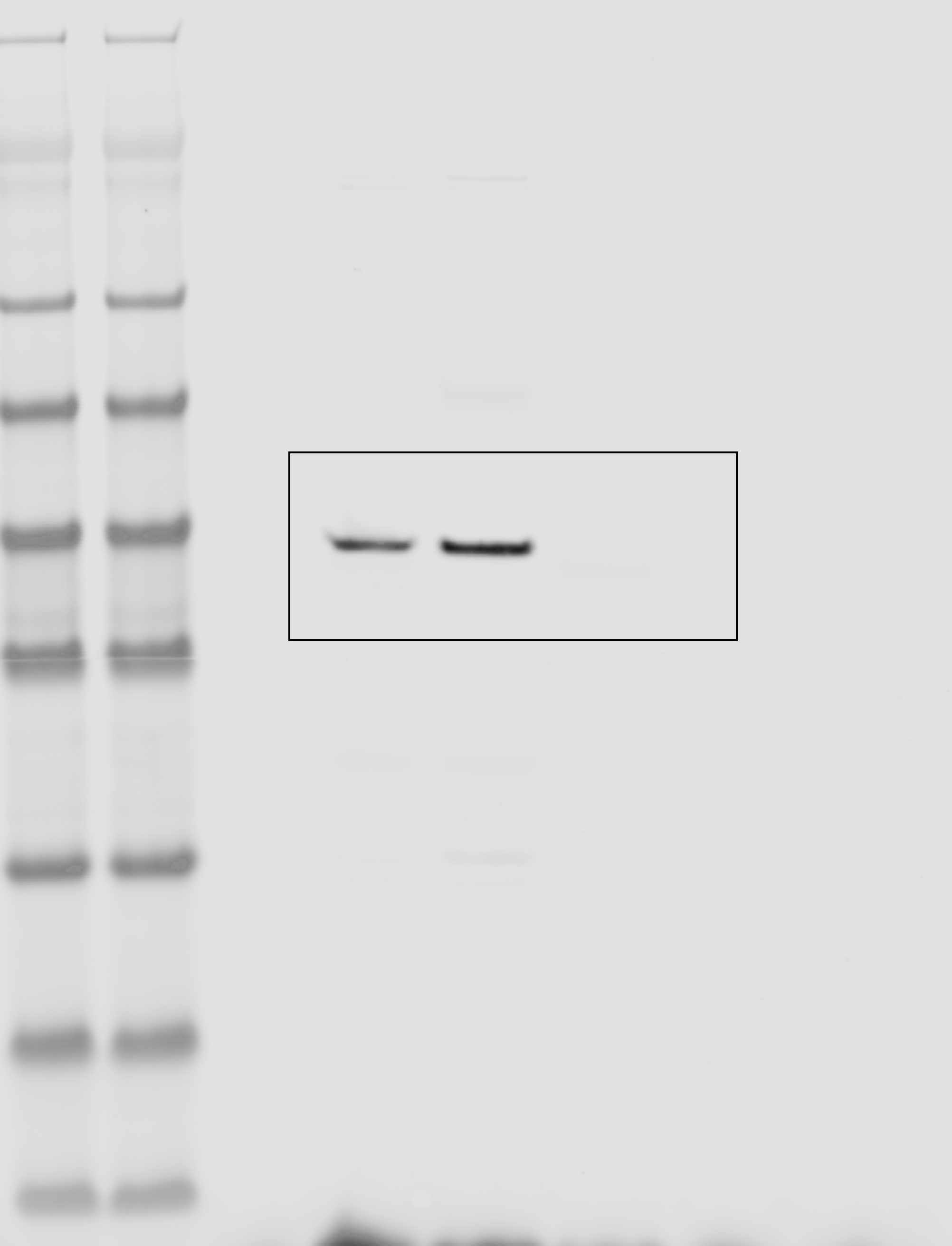

Supplement: Figure 6—figure supplement 1—source data 1. [file elife-89015-fig6-figsupp1-data1.zip › Figure 6-figure supplement 1 blots/C - act OT-I riboP.tif]

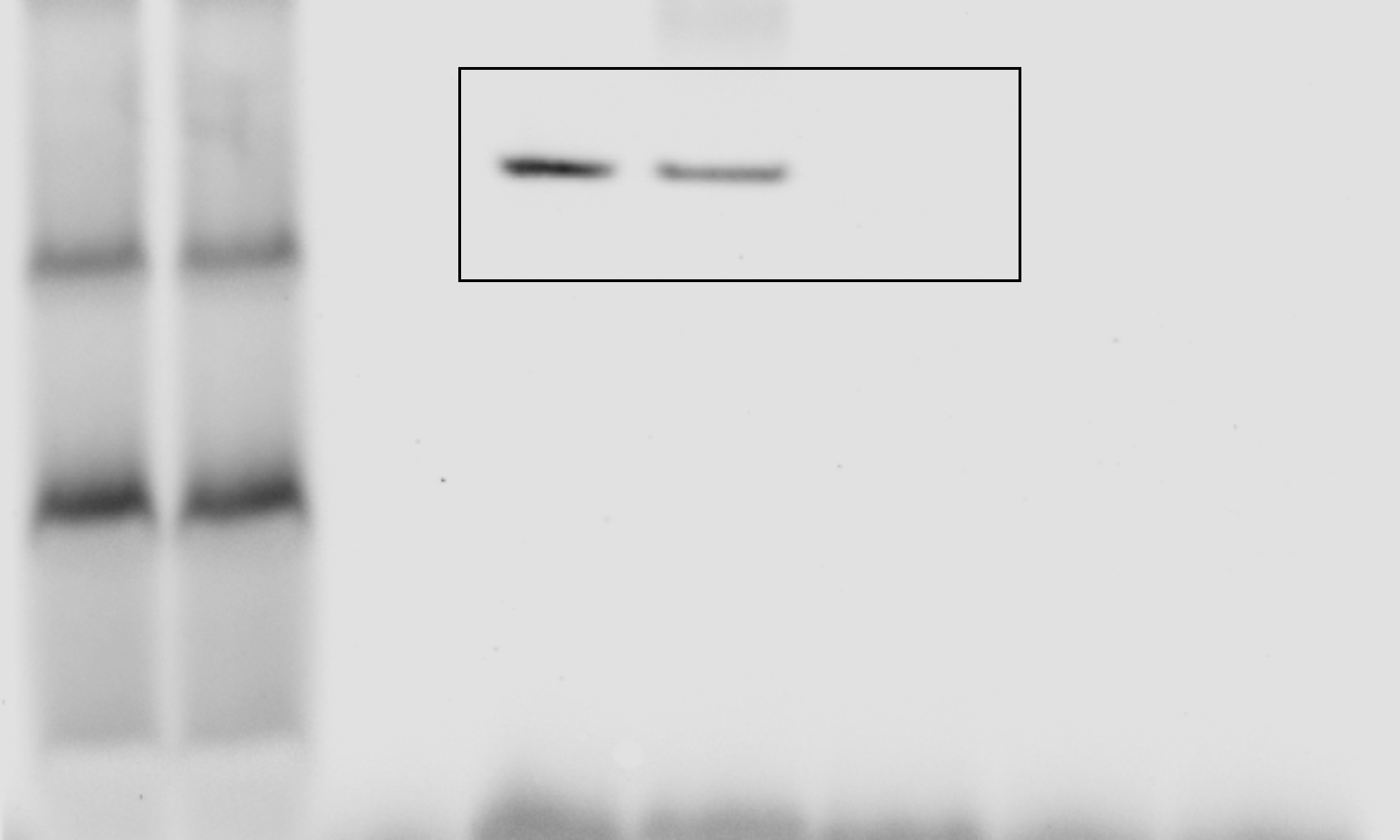

Supplement: Figure 6—figure supplement 1—source data 1. [file elife-89015-fig6-figsupp1-data1.zip › Figure 6-figure supplement 1 blots/C - act OT-I RPL28.tif]

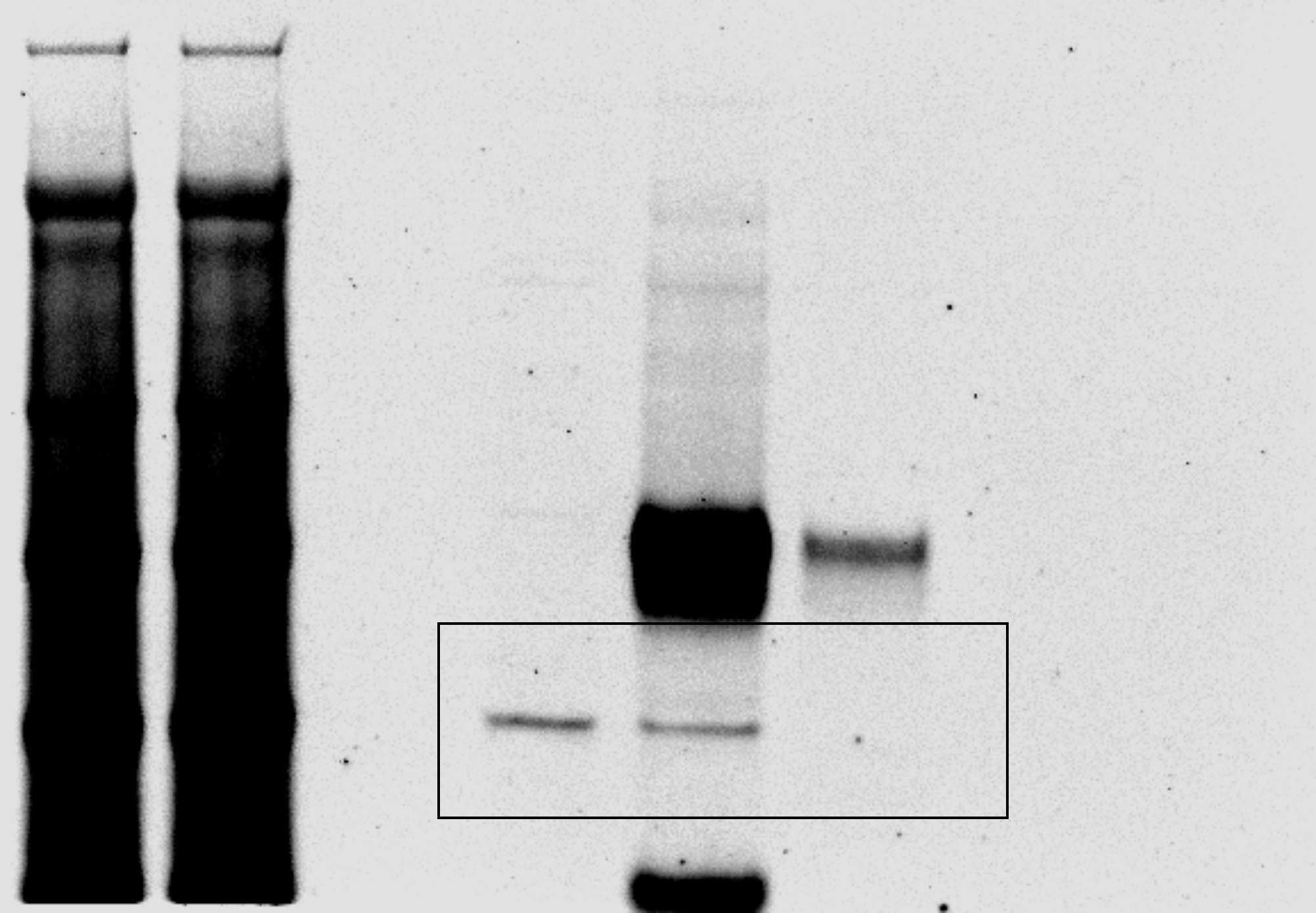

Supplement: Figure 6—figure supplement 1—source data 1. [file elife-89015-fig6-figsupp1-data1.zip › Figure 6-figure supplement 1 blots/C - act OT-I RPL6.tif]

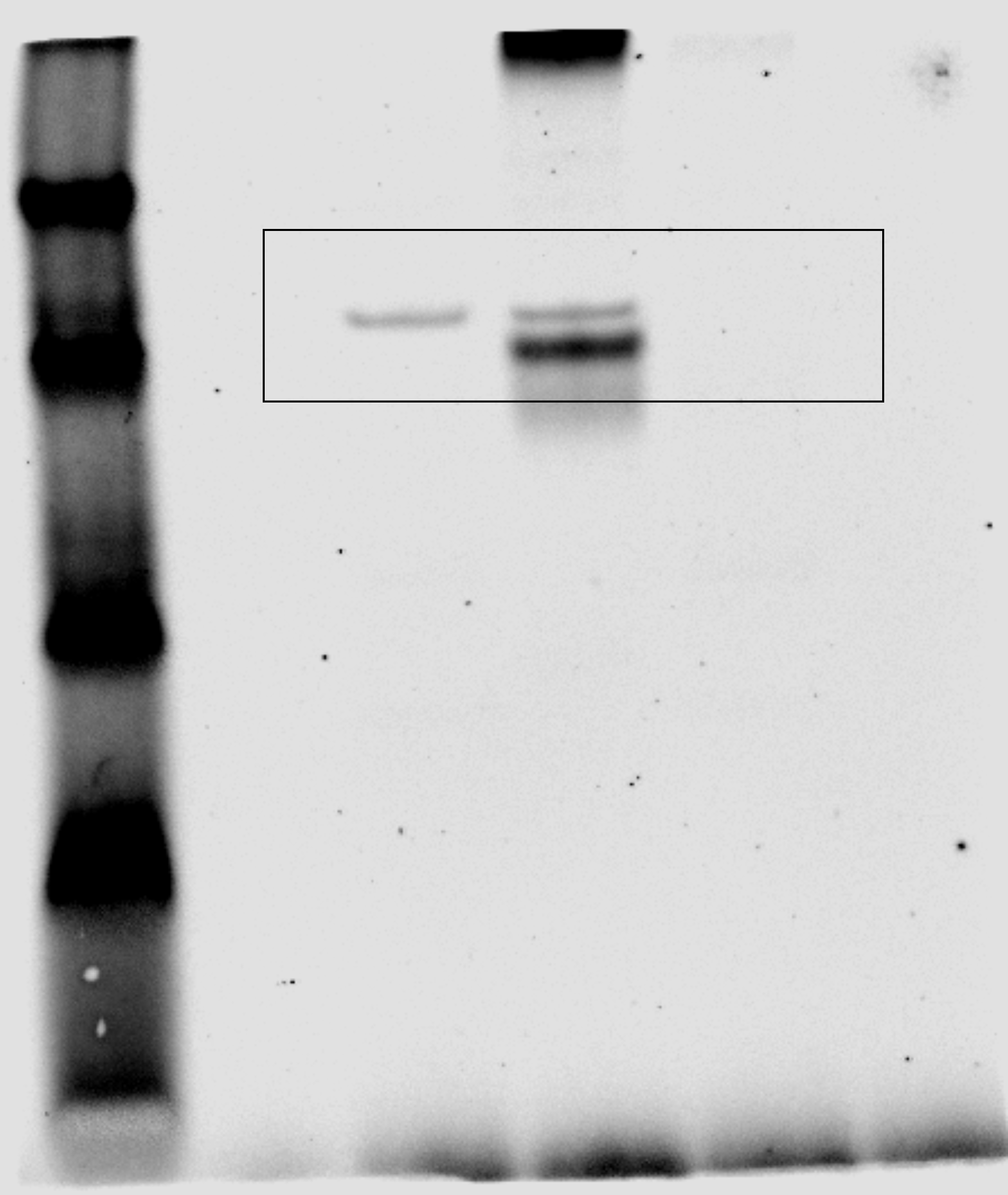

Supplement: Figure 6—figure supplement 1—source data 1. [file elife-89015-fig6-figsupp1-data1.zip › Figure 6-figure supplement 1 blots/C - act OT-I RPS3.tif]

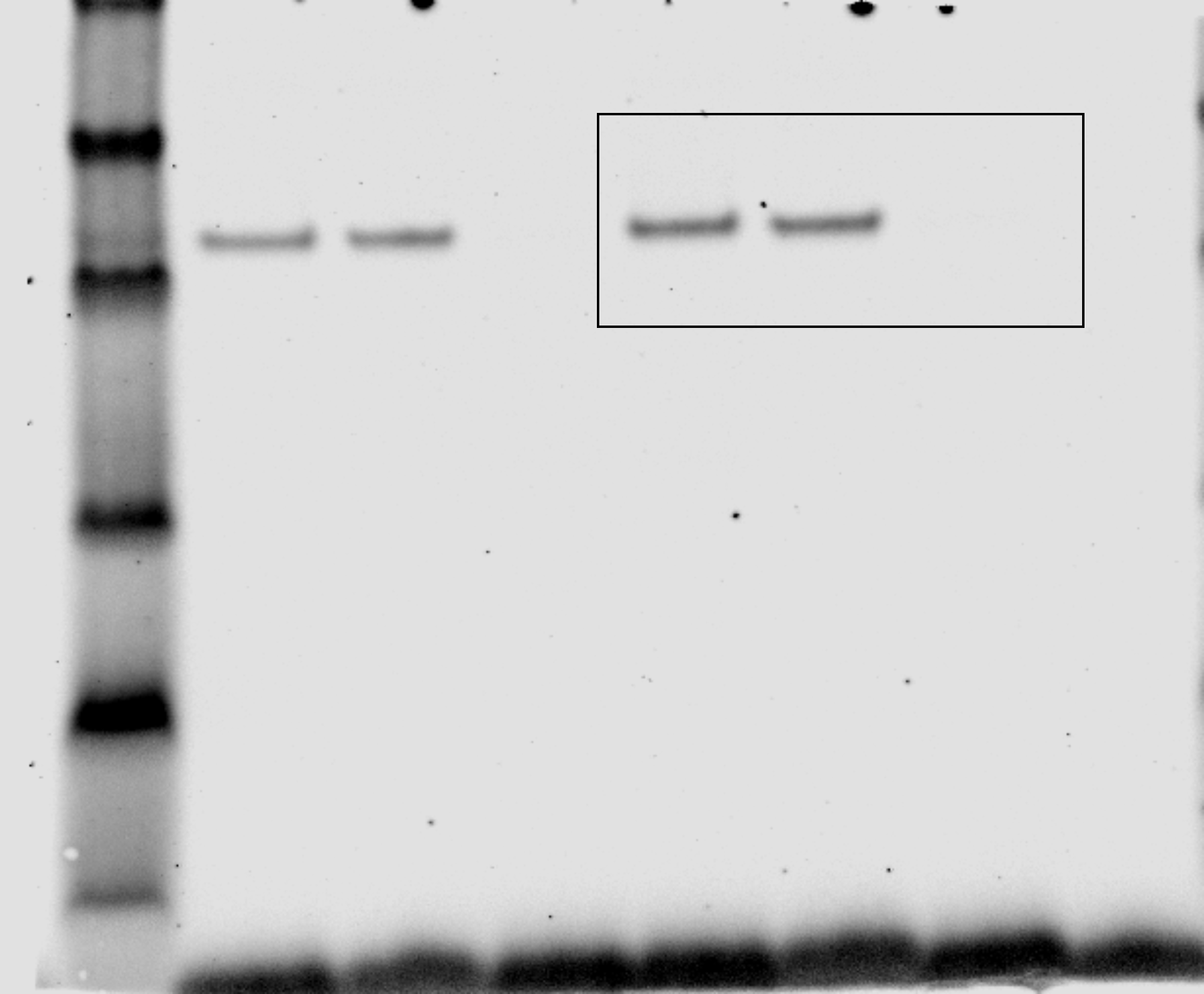

Supplement: Figure 6—figure supplement 1—source data 1. [file elife-89015-fig6-figsupp1-data1.zip › Figure 6-figure supplement 1 blots/C - act OT-I RPS6.tif]

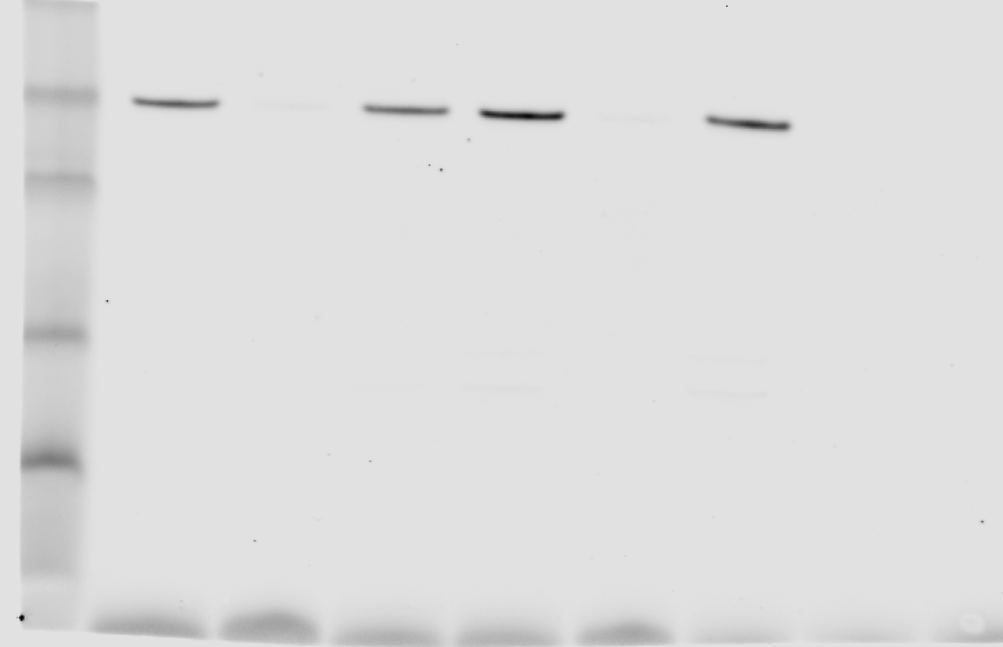

Supplement: Figure 6—figure supplement 1—source data 2. [file elife-89015-fig6-figsupp1-data2.zip › Figure 6-figure supplement 1 raw blots/A - HeLa fibrillarin raw.tif]

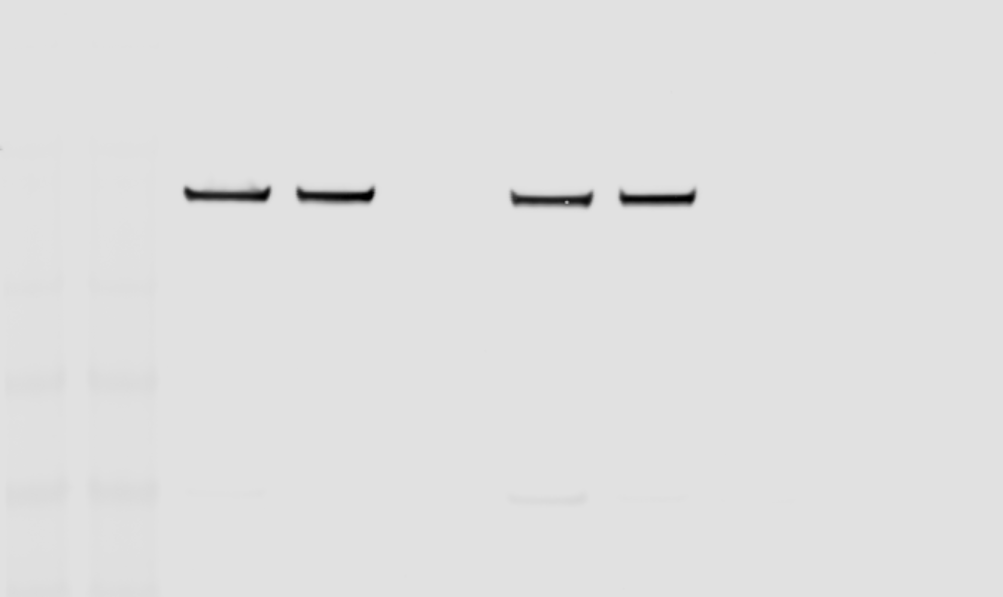

Supplement: Figure 6—figure supplement 1—source data 2. [file elife-89015-fig6-figsupp1-data2.zip › Figure 6-figure supplement 1 raw blots/A - HeLa GRP94 raw.tif]

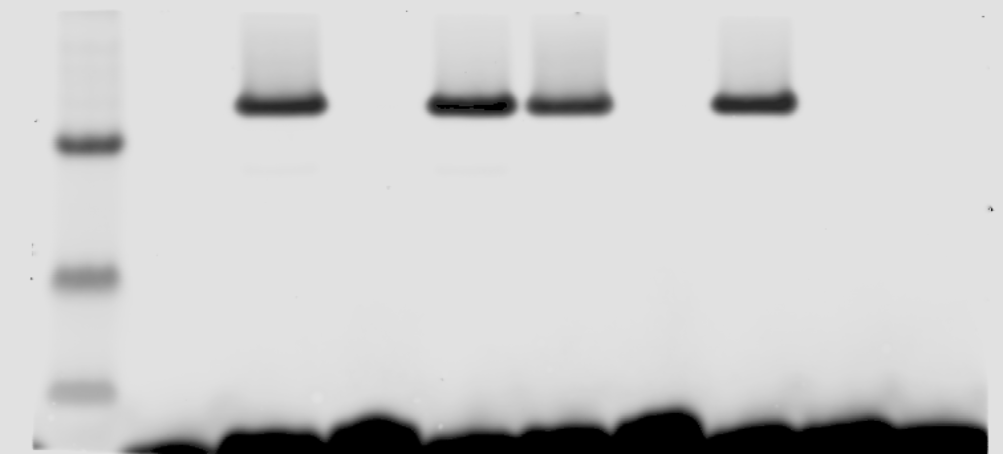

Supplement: Figure 6—figure supplement 1—source data 2. [file elife-89015-fig6-figsupp1-data2.zip › Figure 6-figure supplement 1 raw blots/A - HeLa histone H3 raw.tif]

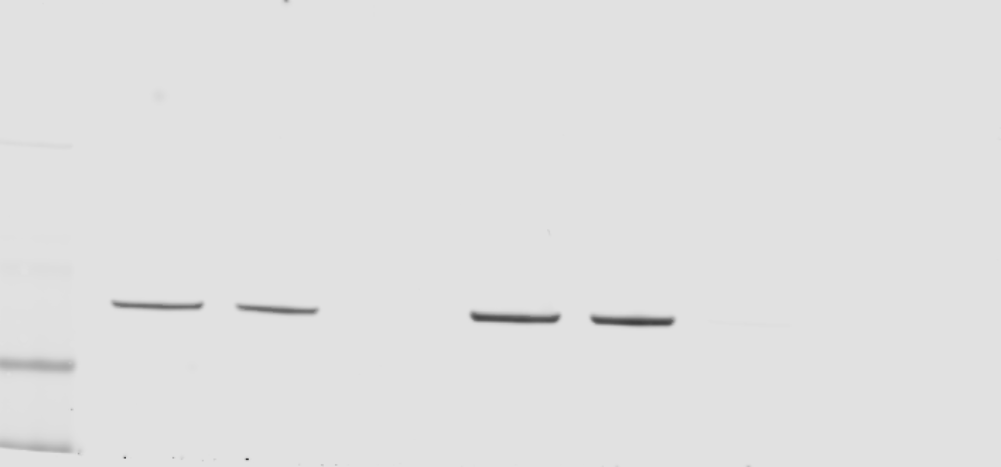

Supplement: Figure 6—figure supplement 1—source data 2. [file elife-89015-fig6-figsupp1-data2.zip › Figure 6-figure supplement 1 raw blots/A - HeLa HSP90 raw.tif]

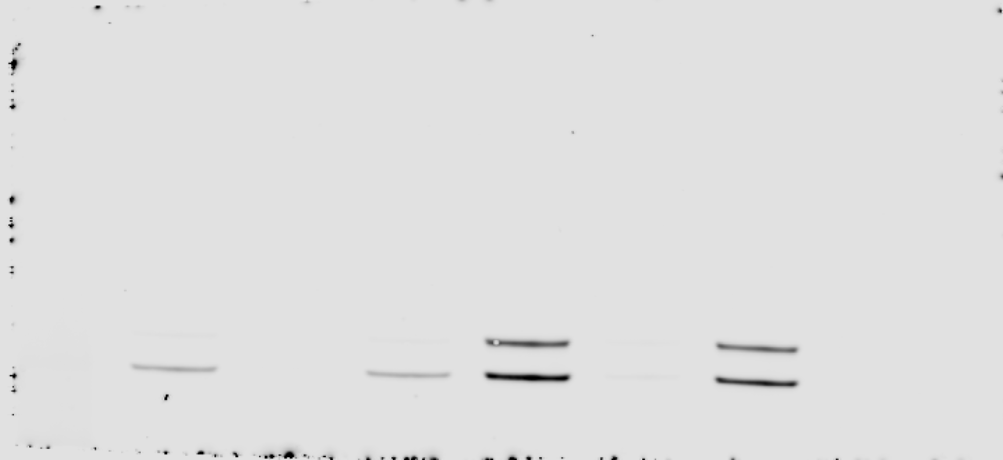

Supplement: Figure 6—figure supplement 1—source data 2. [file elife-89015-fig6-figsupp1-data2.zip › Figure 6-figure supplement 1 raw blots/A - HeLa lamin raw.tif]

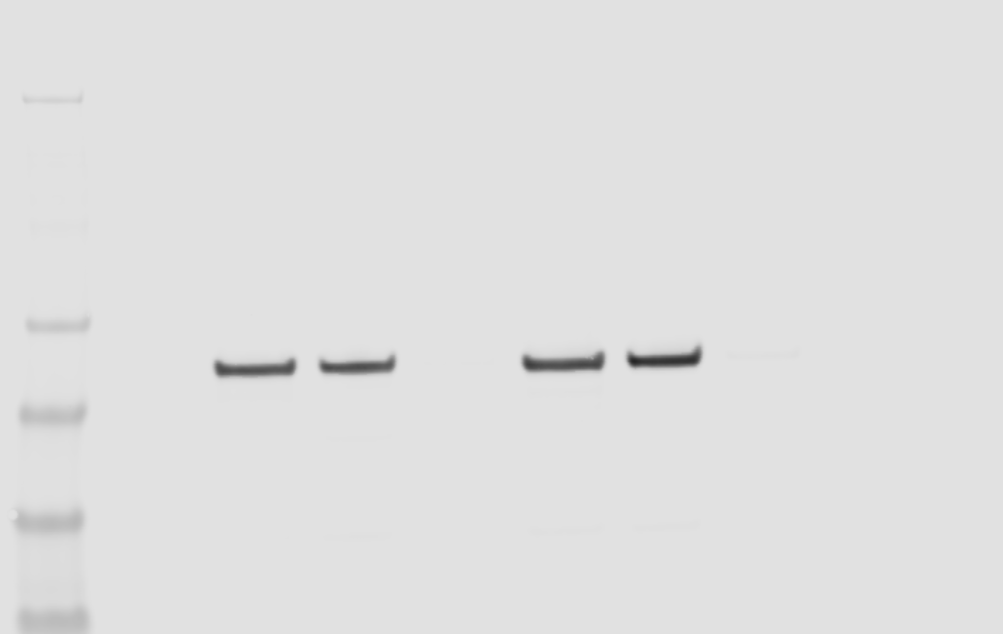

Supplement: Figure 6—figure supplement 1—source data 2. [file elife-89015-fig6-figsupp1-data2.zip › Figure 6-figure supplement 1 raw blots/A - HeLa PDI raw.tif]

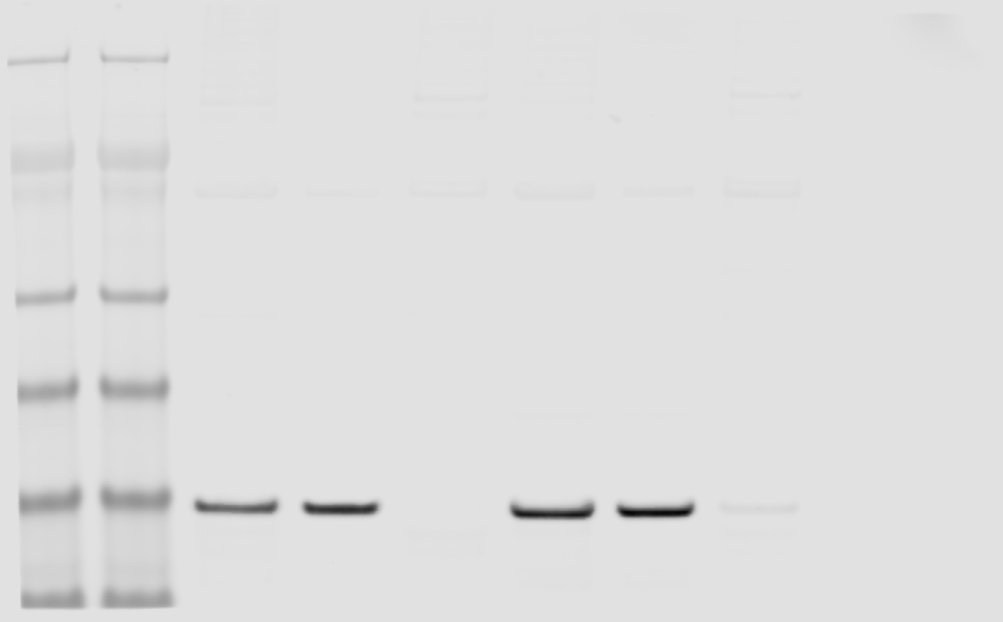

Supplement: Figure 6—figure supplement 1—source data 2. [file elife-89015-fig6-figsupp1-data2.zip › Figure 6-figure supplement 1 raw blots/A - HeLa riboP raw.tif]

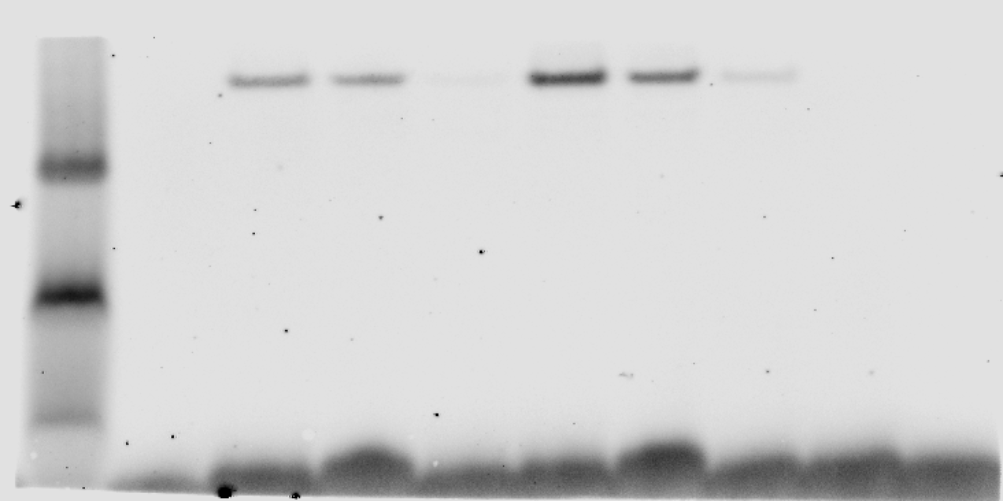

Supplement: Figure 6—figure supplement 1—source data 2. [file elife-89015-fig6-figsupp1-data2.zip › Figure 6-figure supplement 1 raw blots/A - HeLa RPL26 raw.tif]

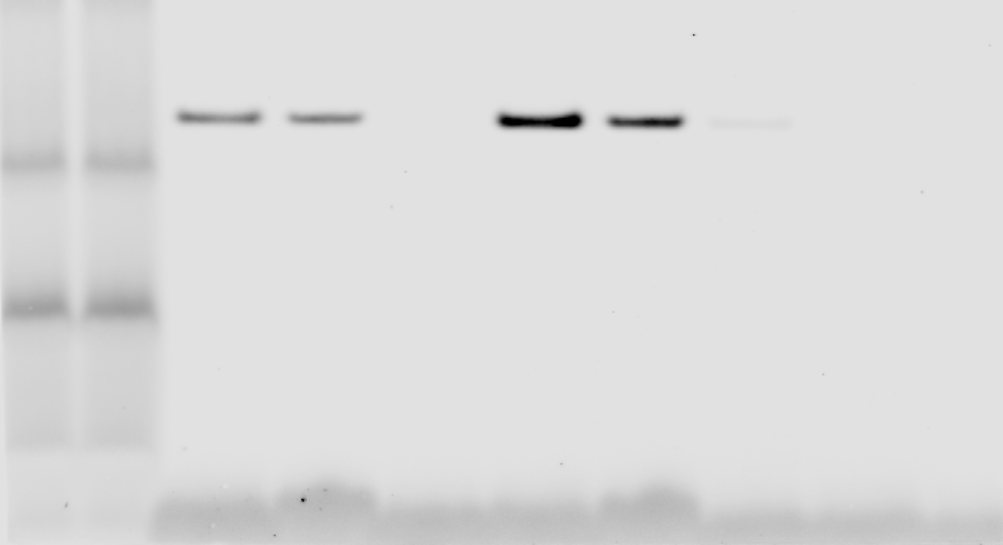

Supplement: Figure 6—figure supplement 1—source data 2. [file elife-89015-fig6-figsupp1-data2.zip › Figure 6-figure supplement 1 raw blots/A - HeLa RPL28 raw.tif]

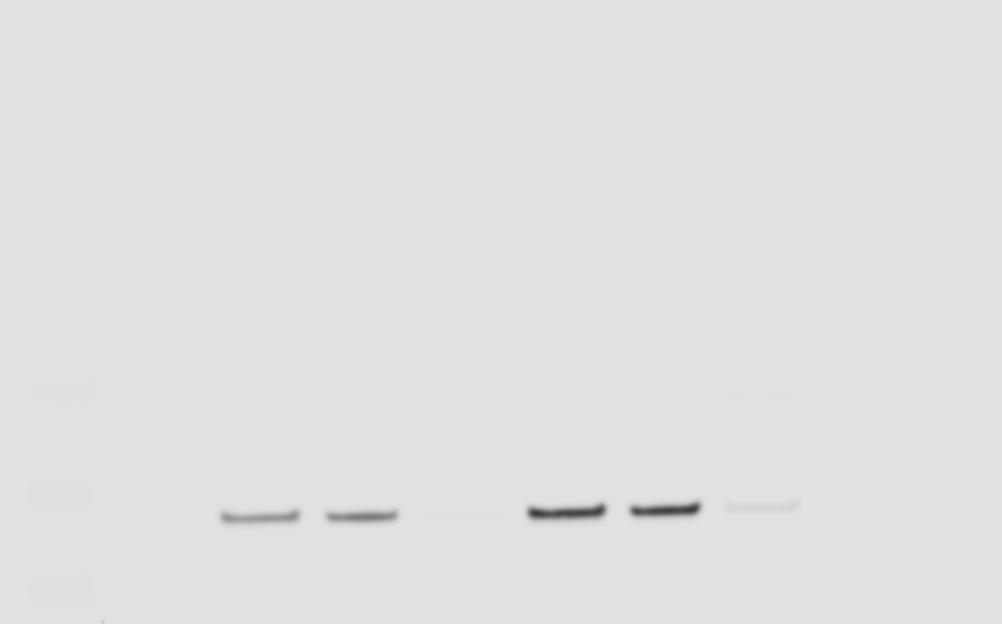

Supplement: Figure 6—figure supplement 1—source data 2. [file elife-89015-fig6-figsupp1-data2.zip › Figure 6-figure supplement 1 raw blots/A - HeLa RPL5 raw.tif]

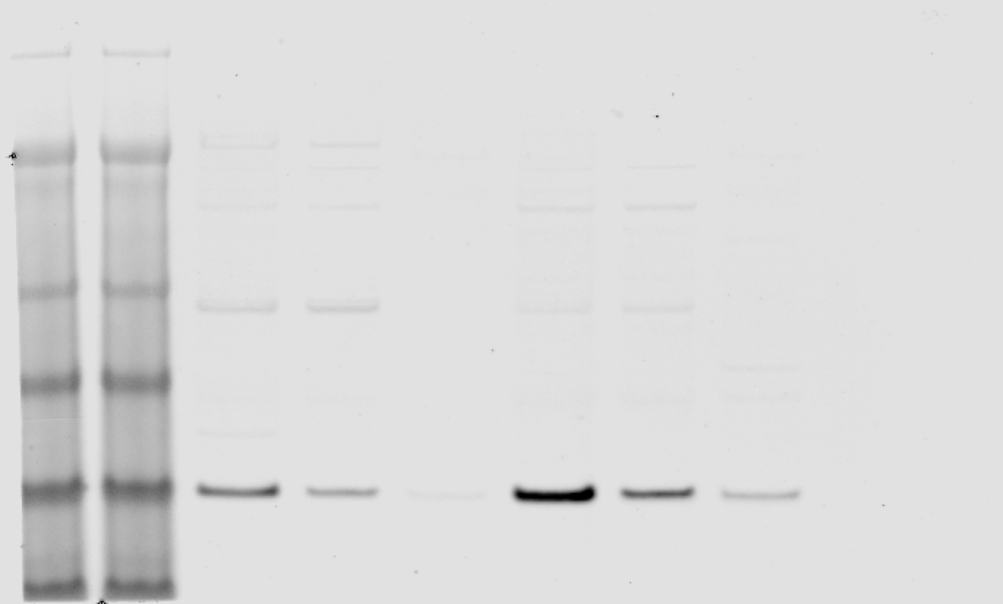

Supplement: Figure 6—figure supplement 1—source data 2. [file elife-89015-fig6-figsupp1-data2.zip › Figure 6-figure supplement 1 raw blots/A - HeLa RPL6 raw.tif]

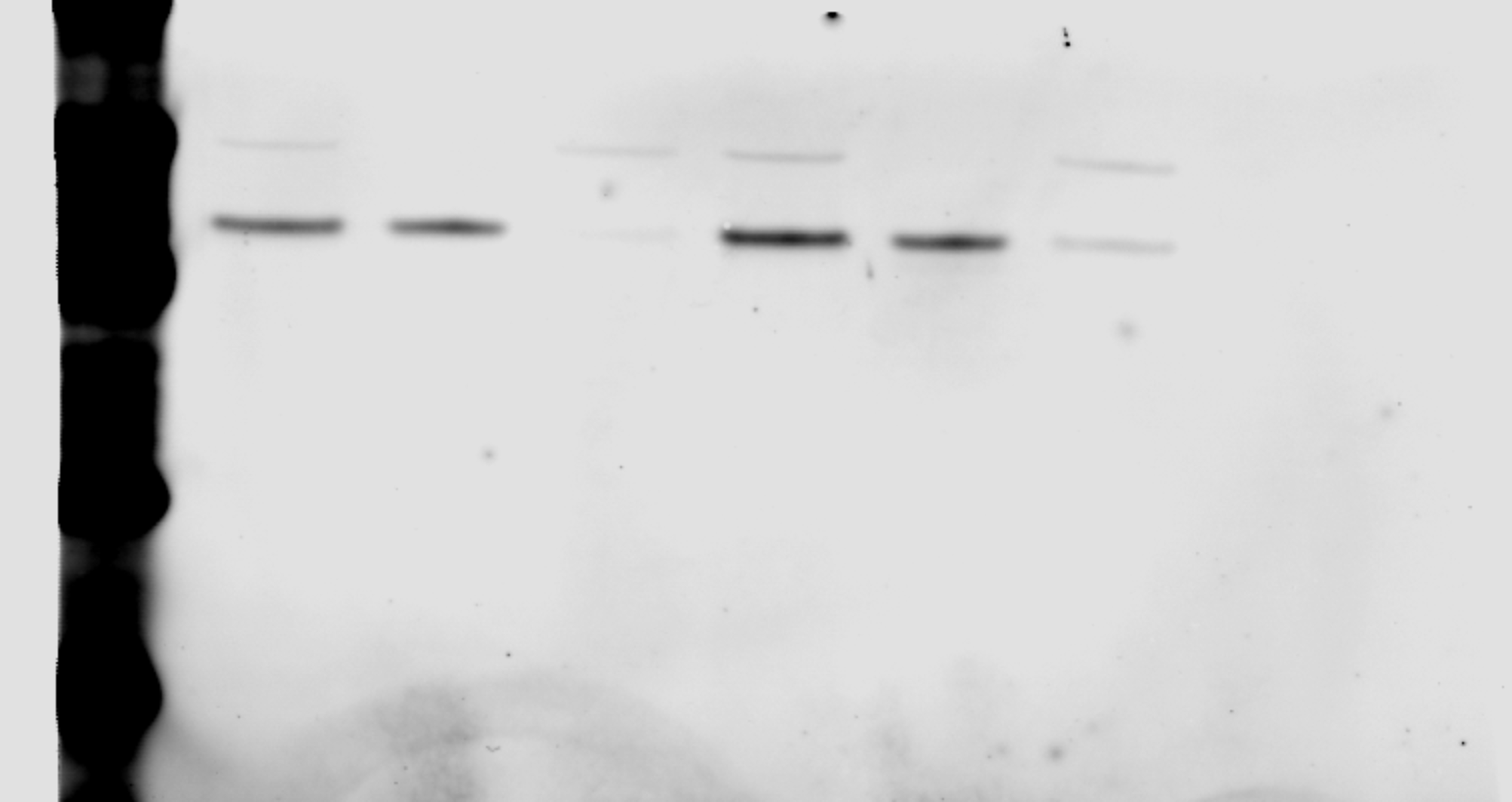

Supplement: Figure 6—figure supplement 1—source data 2. [file elife-89015-fig6-figsupp1-data2.zip › Figure 6-figure supplement 1 raw blots/A - HeLa RPS6 raw.tif]

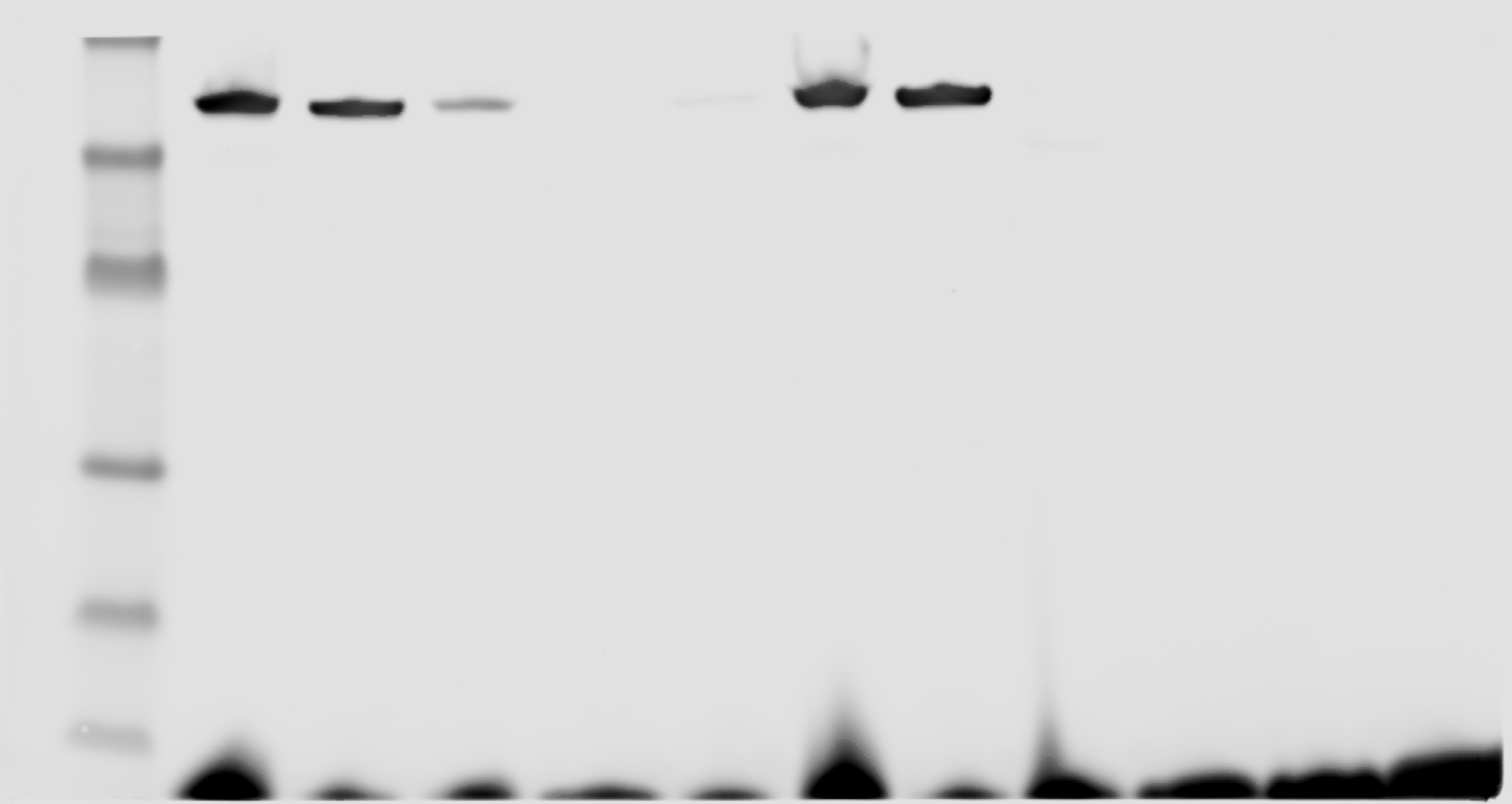

Supplement: Figure 6—figure supplement 1—source data 2. [file elife-89015-fig6-figsupp1-data2.zip › Figure 6-figure supplement 1 raw blots/B - resting OT-I actin raw.tif]

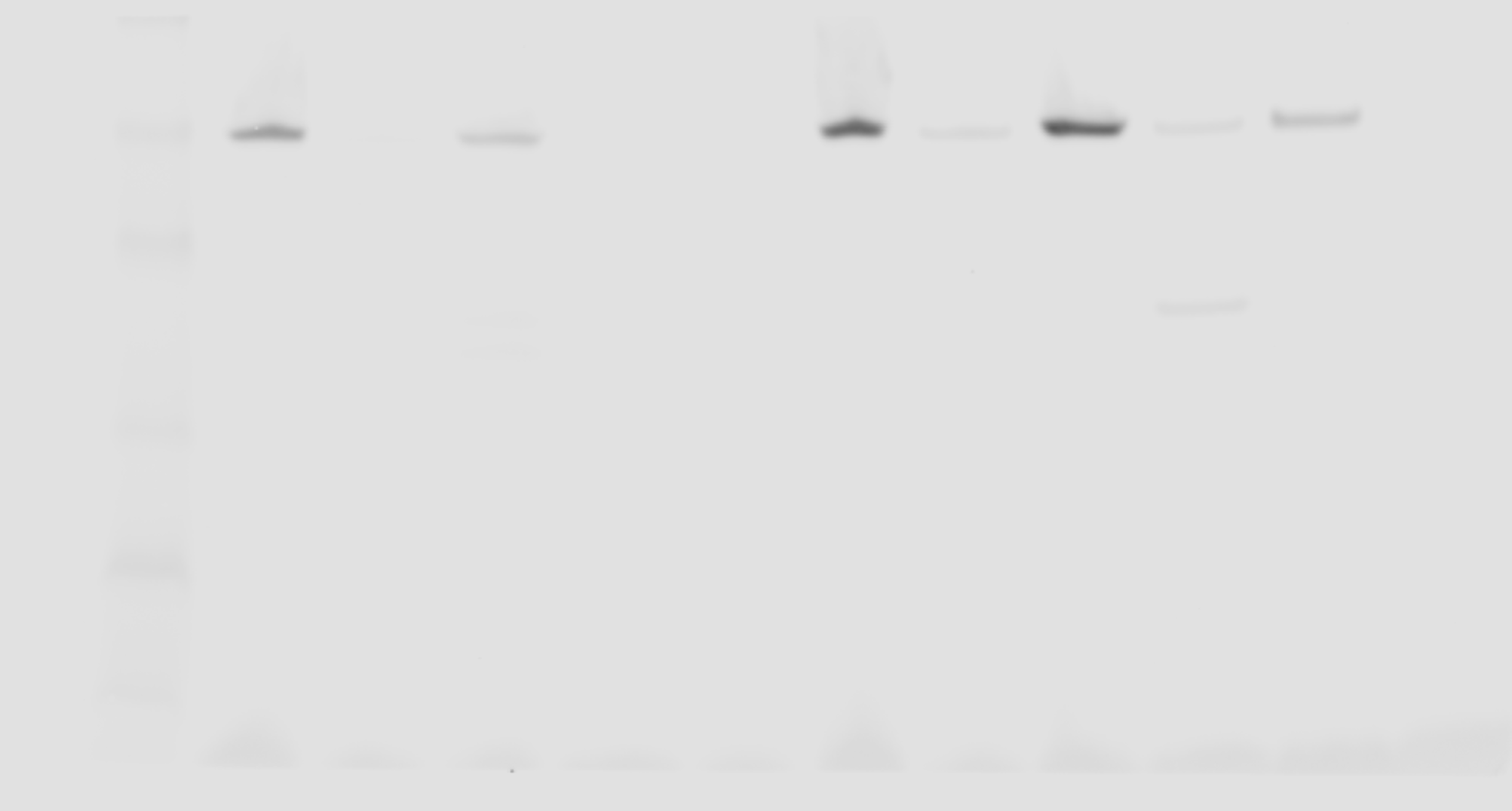

Supplement: Figure 6—figure supplement 1—source data 2. [file elife-89015-fig6-figsupp1-data2.zip › Figure 6-figure supplement 1 raw blots/B - resting OT-I fibrillarin raw.tif]

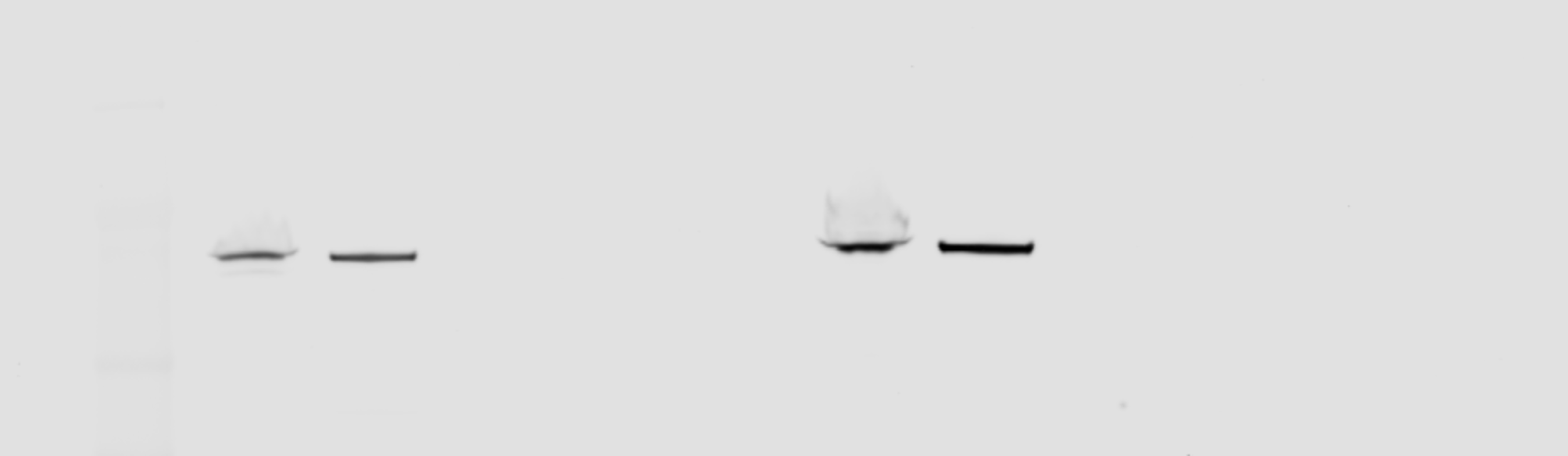

Supplement: Figure 6—figure supplement 1—source data 2. [file elife-89015-fig6-figsupp1-data2.zip › Figure 6-figure supplement 1 raw blots/B - resting OT-I GRP94 raw.tif]

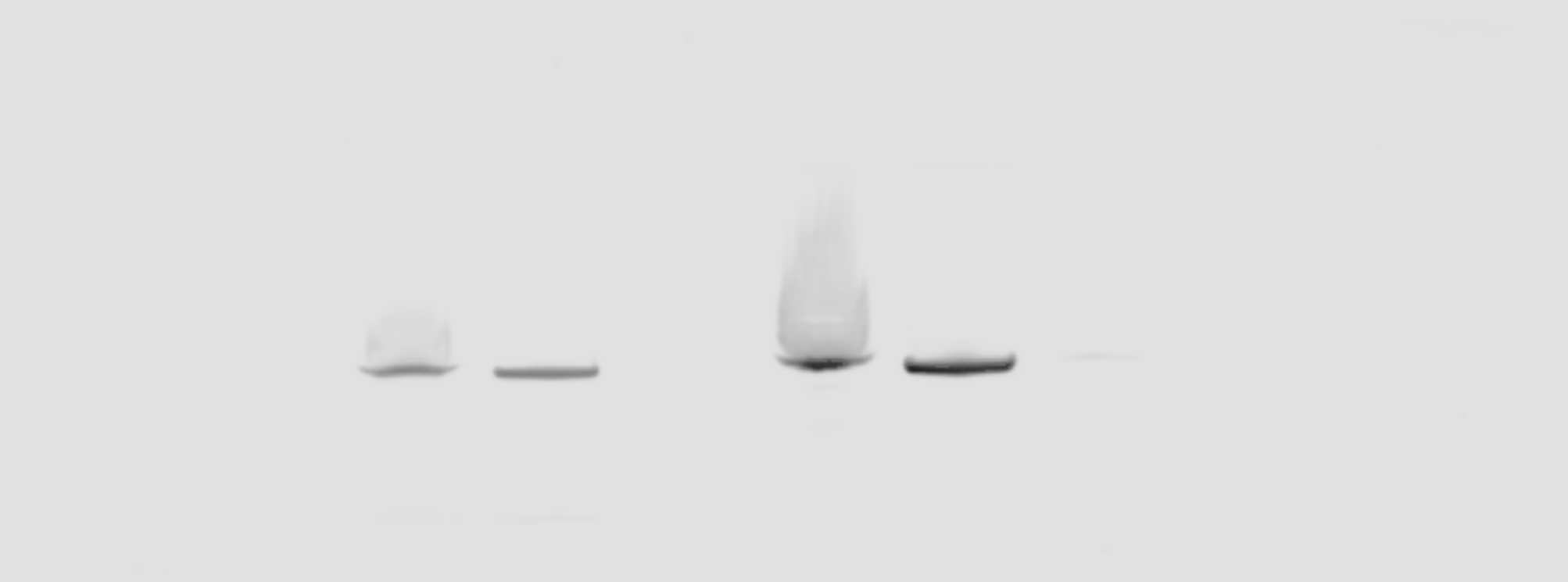

Supplement: Figure 6—figure supplement 1—source data 2. [file elife-89015-fig6-figsupp1-data2.zip › Figure 6-figure supplement 1 raw blots/B - resting OT-I HSP90 raw.tif]

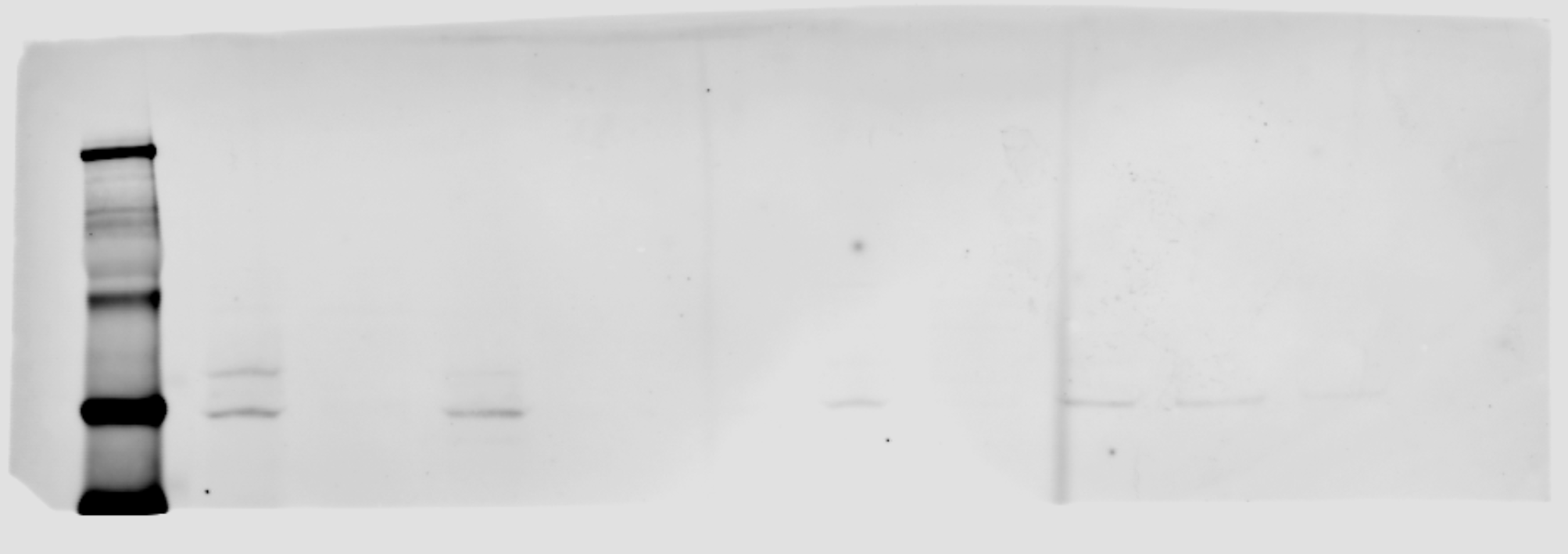

Supplement: Figure 6—figure supplement 1—source data 2. [file elife-89015-fig6-figsupp1-data2.zip › Figure 6-figure supplement 1 raw blots/B - resting OT-I lamin raw.tif]

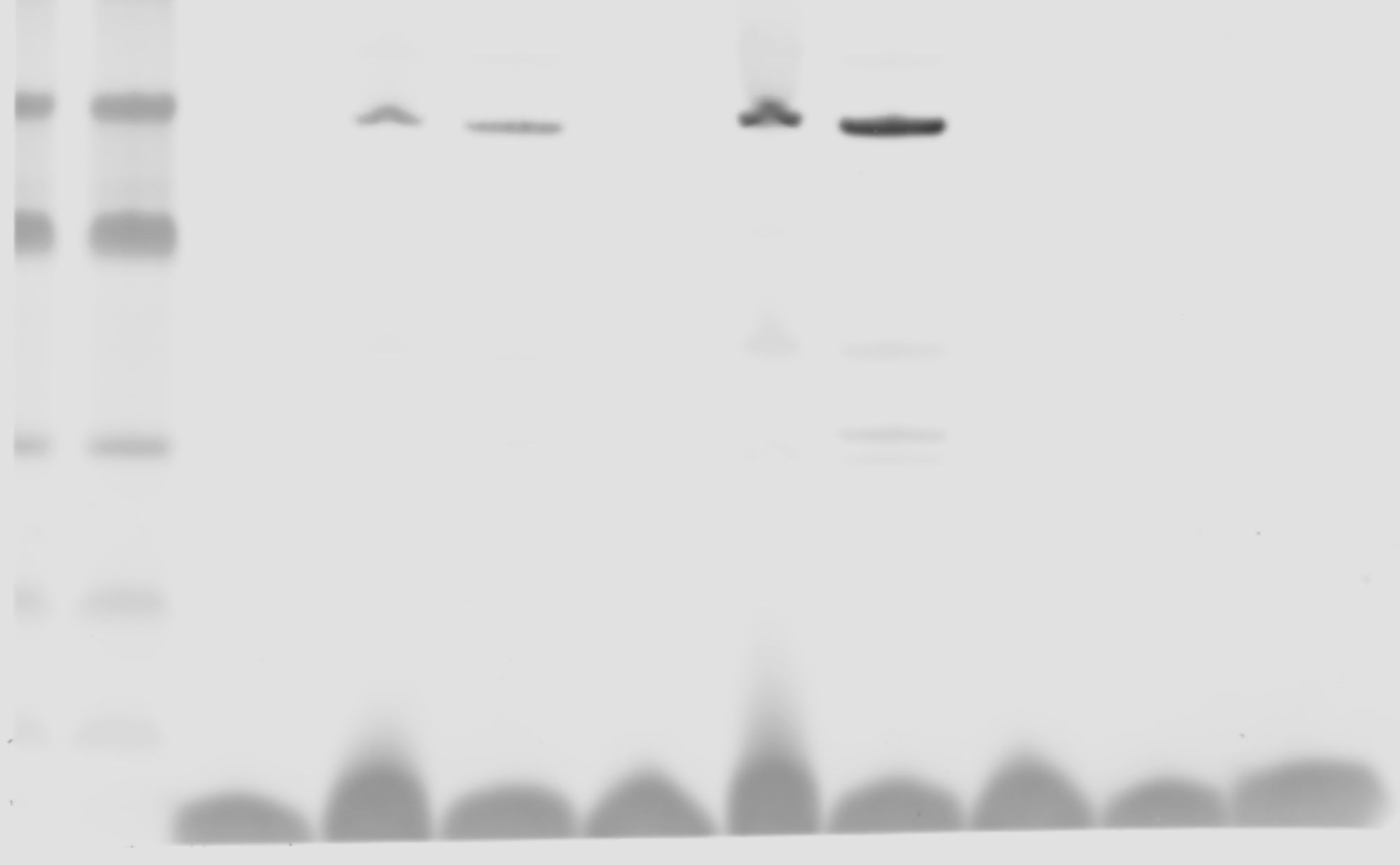

Supplement: Figure 6—figure supplement 1—source data 2. [file elife-89015-fig6-figsupp1-data2.zip › Figure 6-figure supplement 1 raw blots/B - resting OT-I riboP raw.tif]

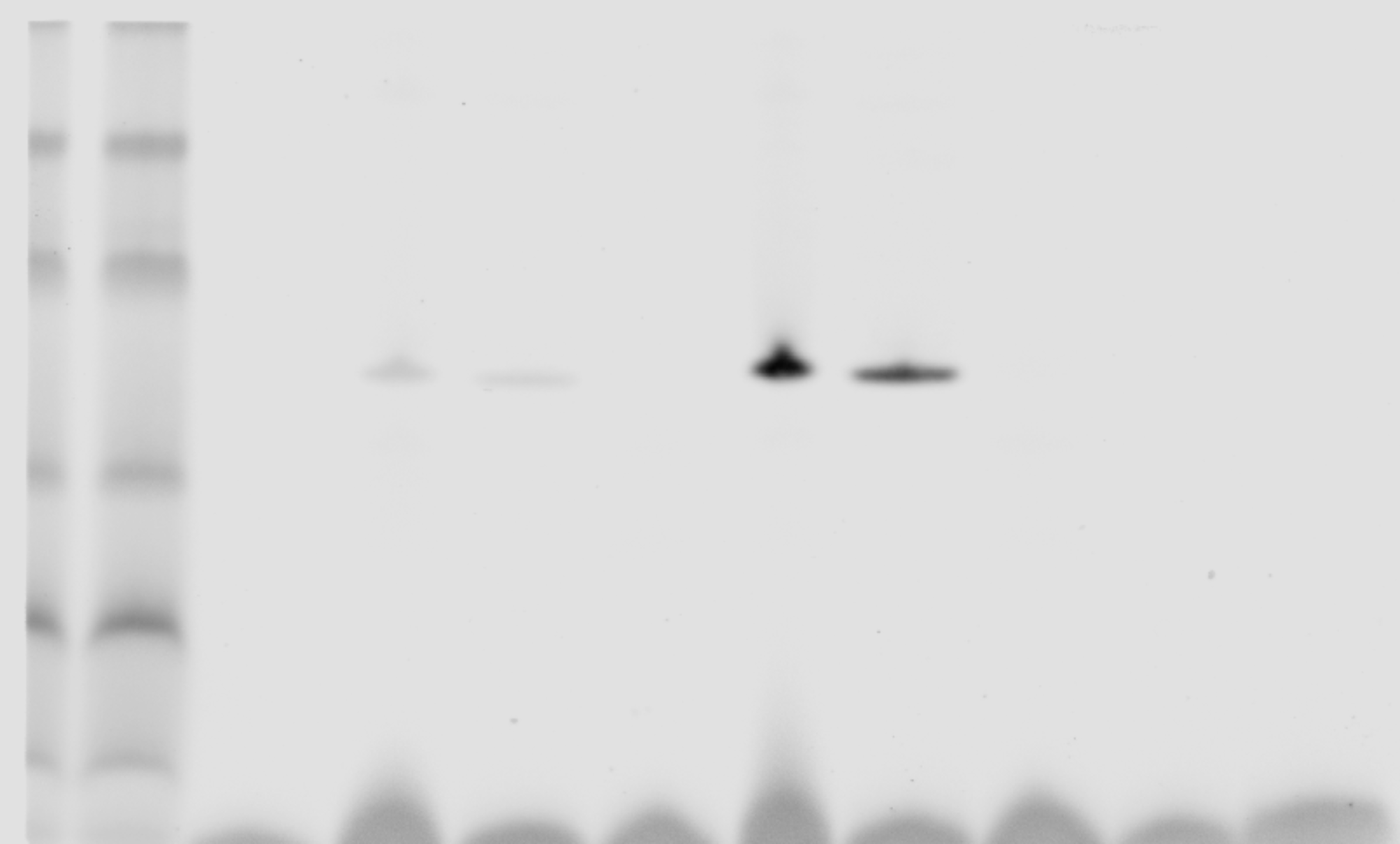

Supplement: Figure 6—figure supplement 1—source data 2. [file elife-89015-fig6-figsupp1-data2.zip › Figure 6-figure supplement 1 raw blots/B - resting OT-I RPL26 raw.tif]

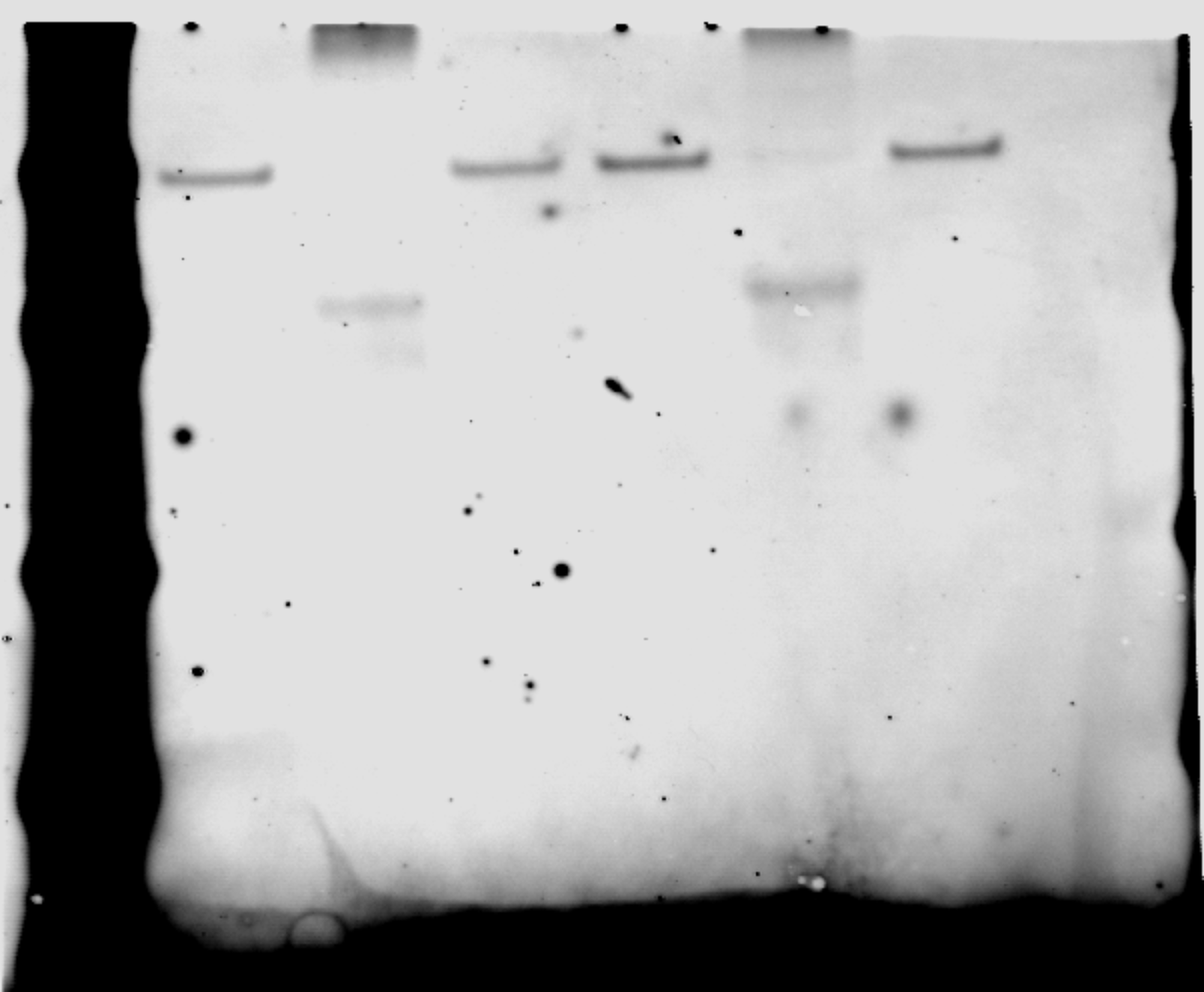

Supplement: Figure 6—figure supplement 1—source data 2. [file elife-89015-fig6-figsupp1-data2.zip › Figure 6-figure supplement 1 raw blots/C - act OT-I fibrillarin raw.tif]

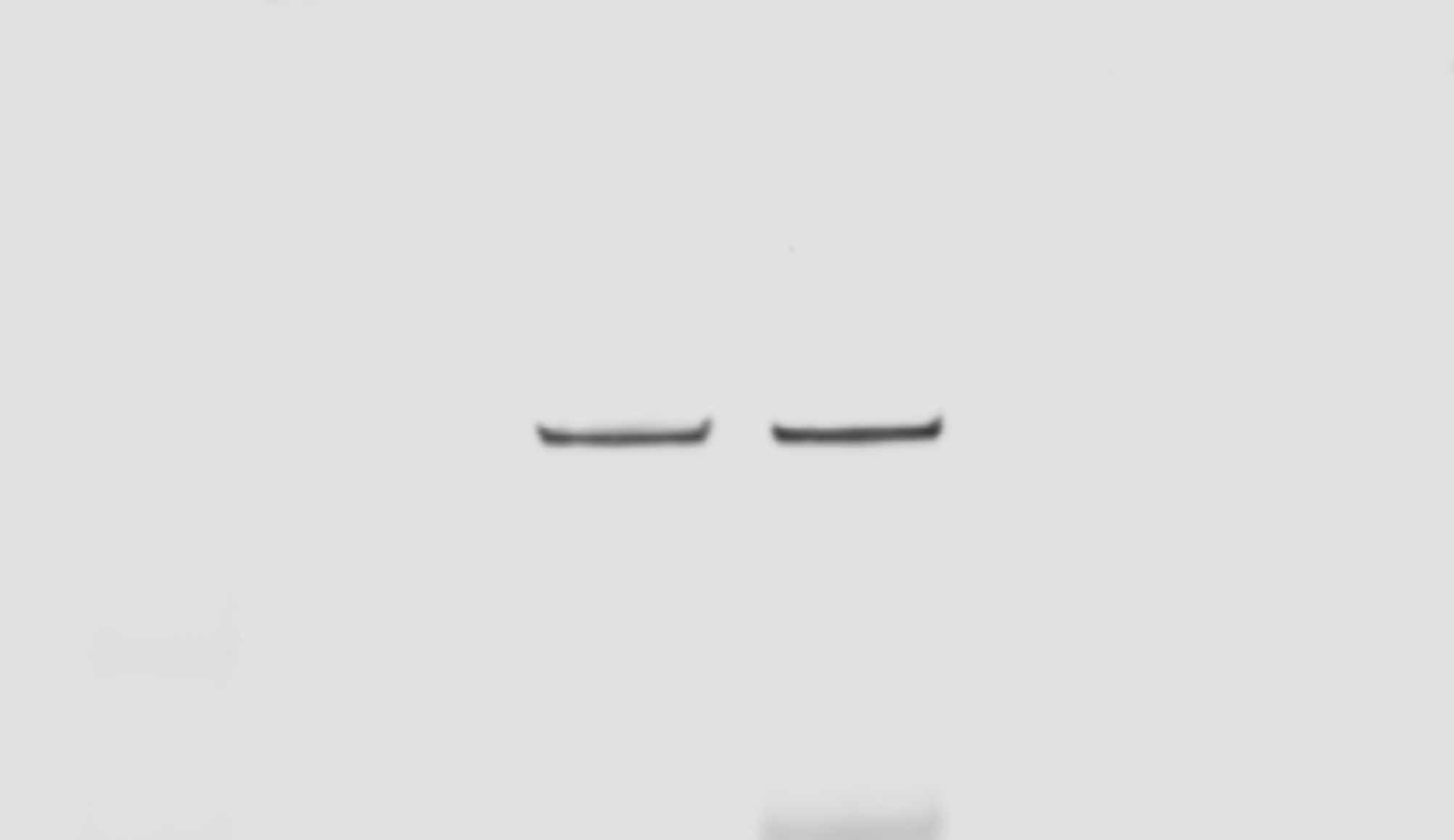

Supplement: Figure 6—figure supplement 1—source data 2. [file elife-89015-fig6-figsupp1-data2.zip › Figure 6-figure supplement 1 raw blots/C - act OT-I GRP94 raw.tif]

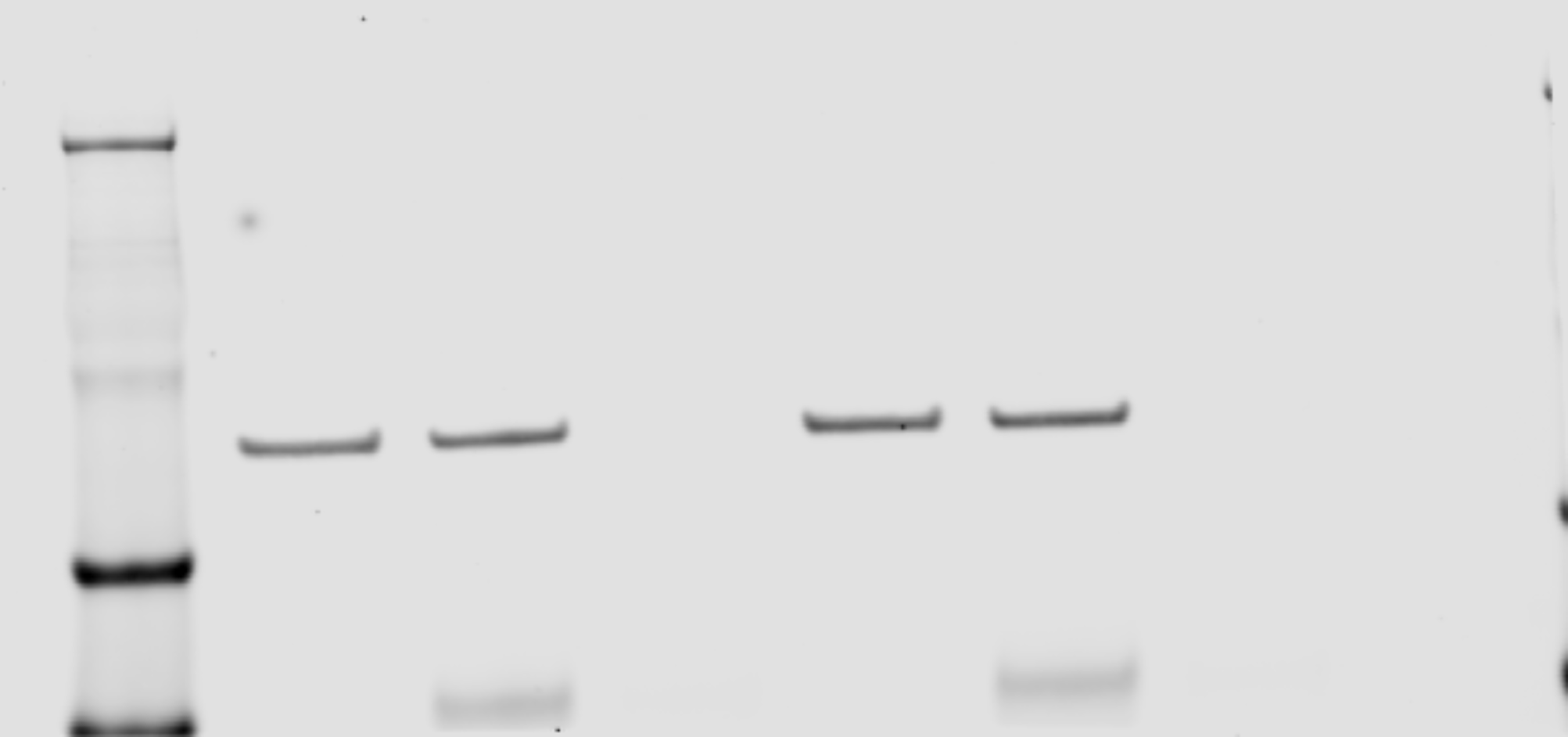

Supplement: Figure 6—figure supplement 1—source data 2. [file elife-89015-fig6-figsupp1-data2.zip › Figure 6-figure supplement 1 raw blots/C - act OT-I HSP90 raw.tif]

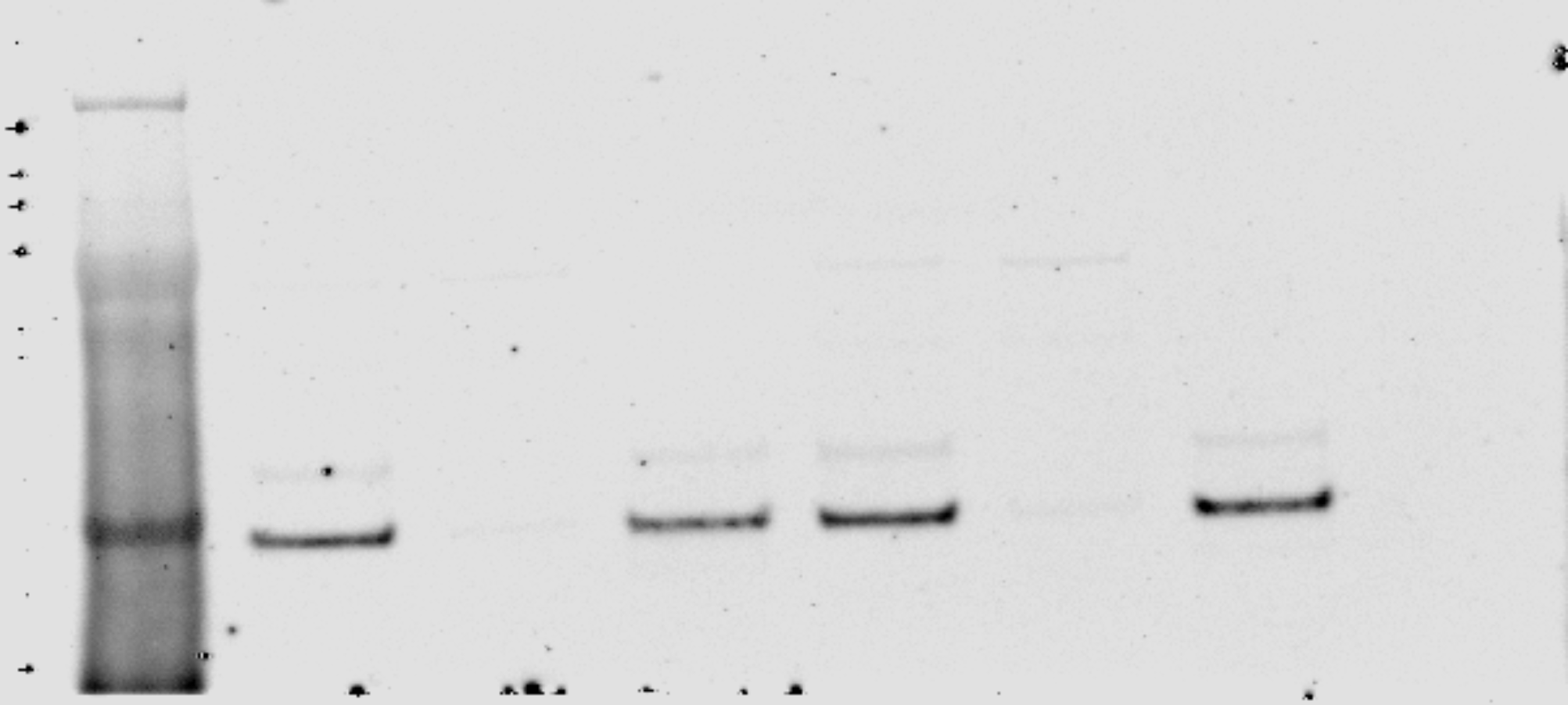

Supplement: Figure 6—figure supplement 1—source data 2. [file elife-89015-fig6-figsupp1-data2.zip › Figure 6-figure supplement 1 raw blots/C - act OT-I lamin raw.tif]

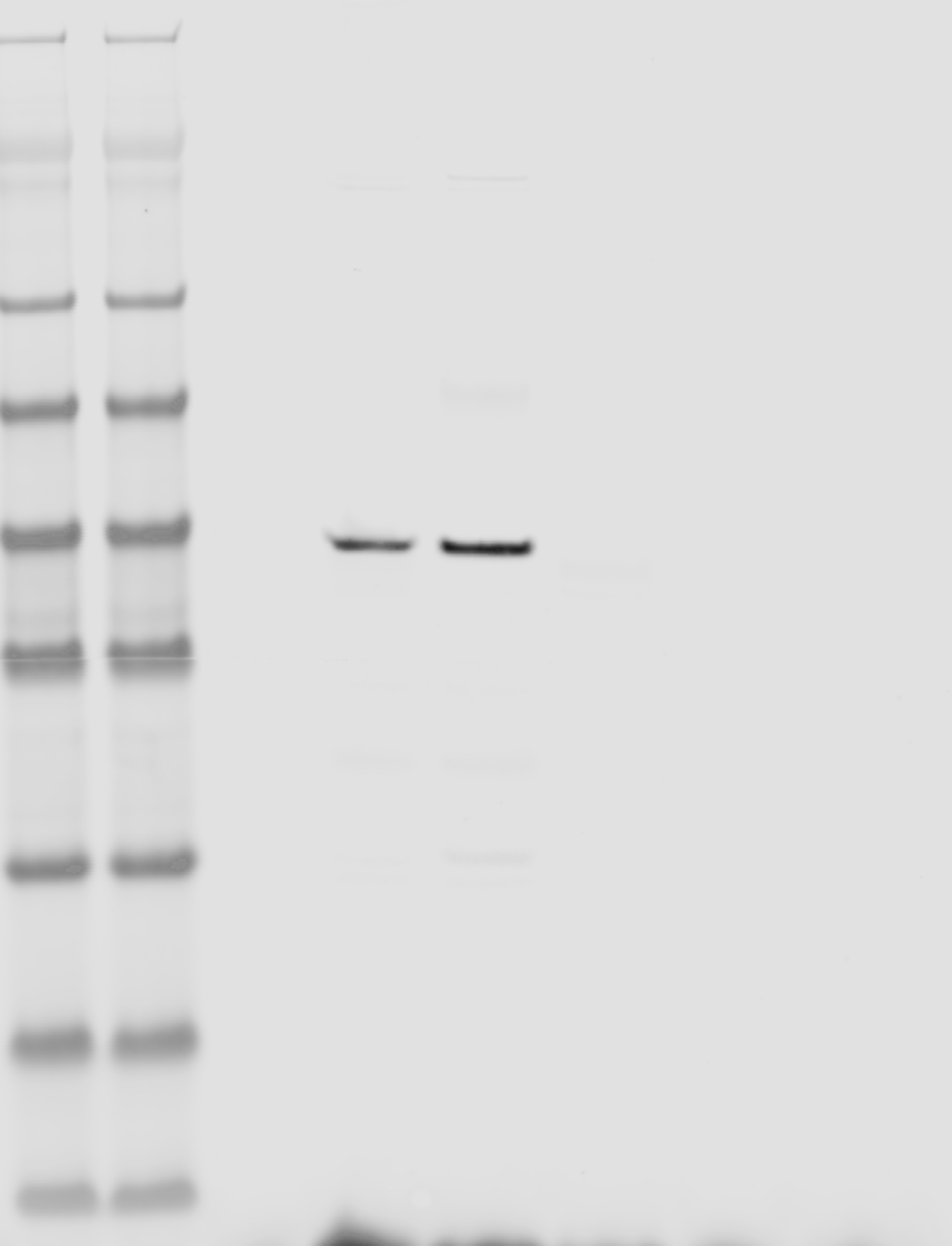

Supplement: Figure 6—figure supplement 1—source data 2. [file elife-89015-fig6-figsupp1-data2.zip › Figure 6-figure supplement 1 raw blots/C - act OT-I riboP raw.tif]

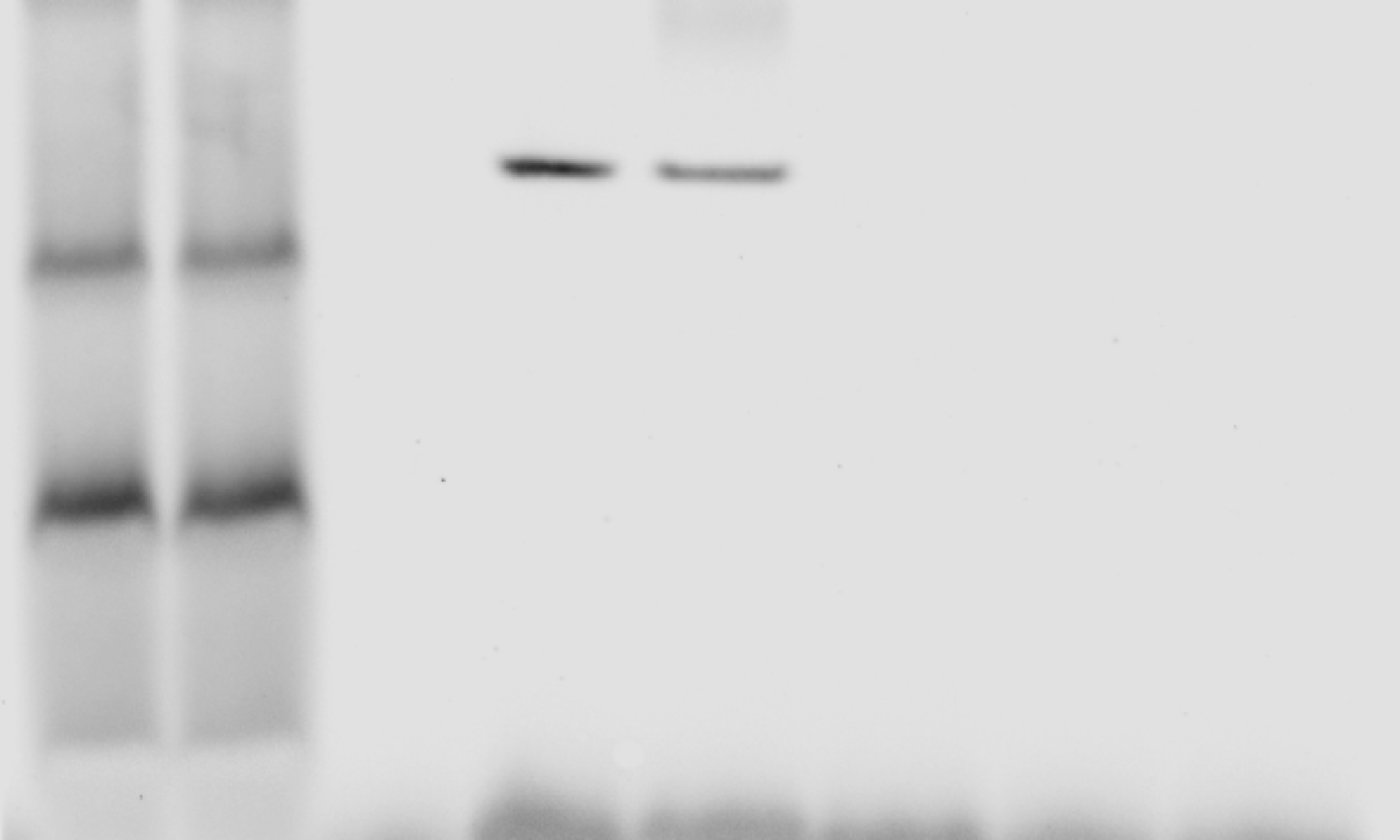

Supplement: Figure 6—figure supplement 1—source data 2. [file elife-89015-fig6-figsupp1-data2.zip › Figure 6-figure supplement 1 raw blots/C - act OT-I RPL28 raw.tif]

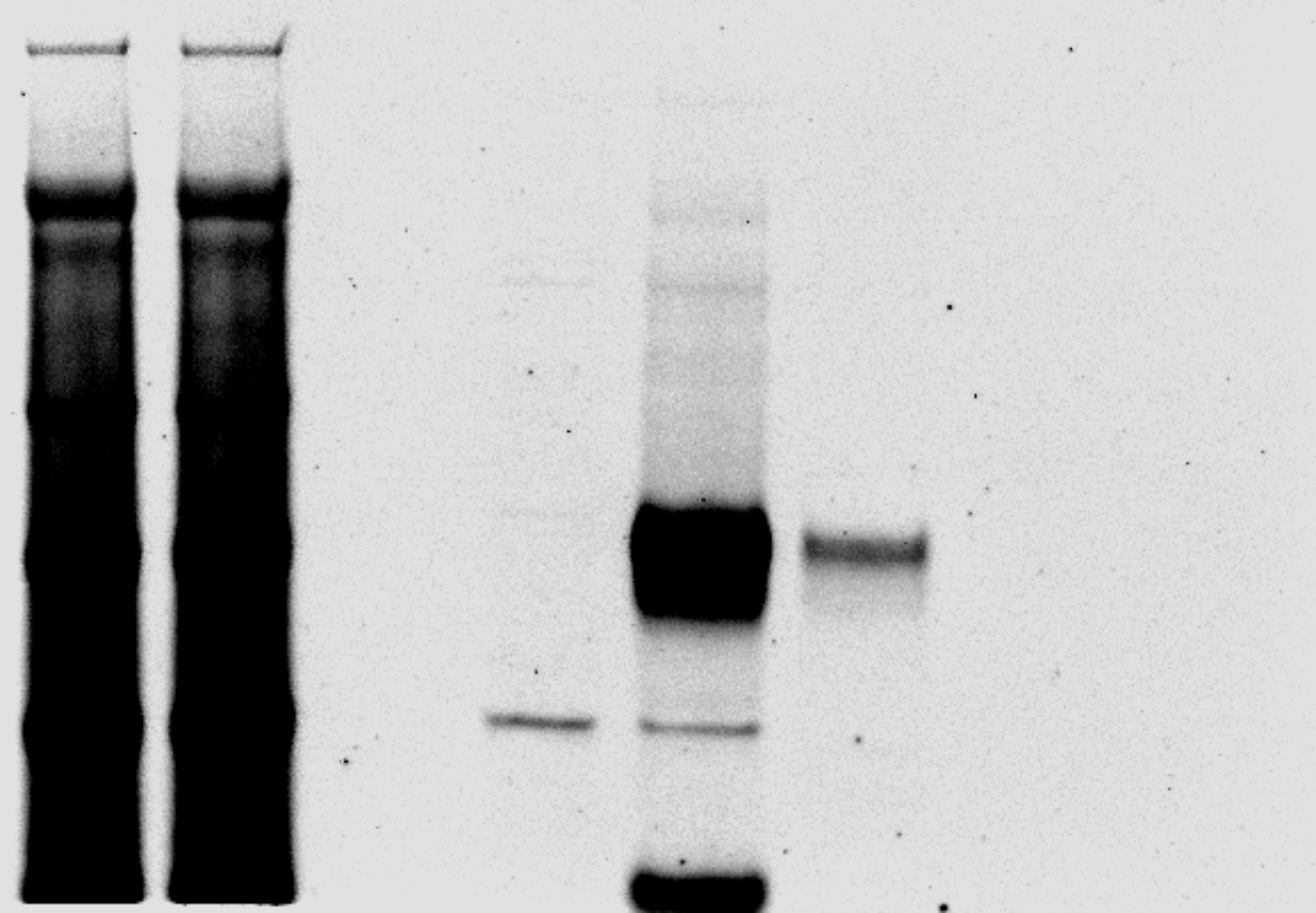

Supplement: Figure 6—figure supplement 1—source data 2. [file elife-89015-fig6-figsupp1-data2.zip › Figure 6-figure supplement 1 raw blots/C - act OT-I RPL6 raw.tif]

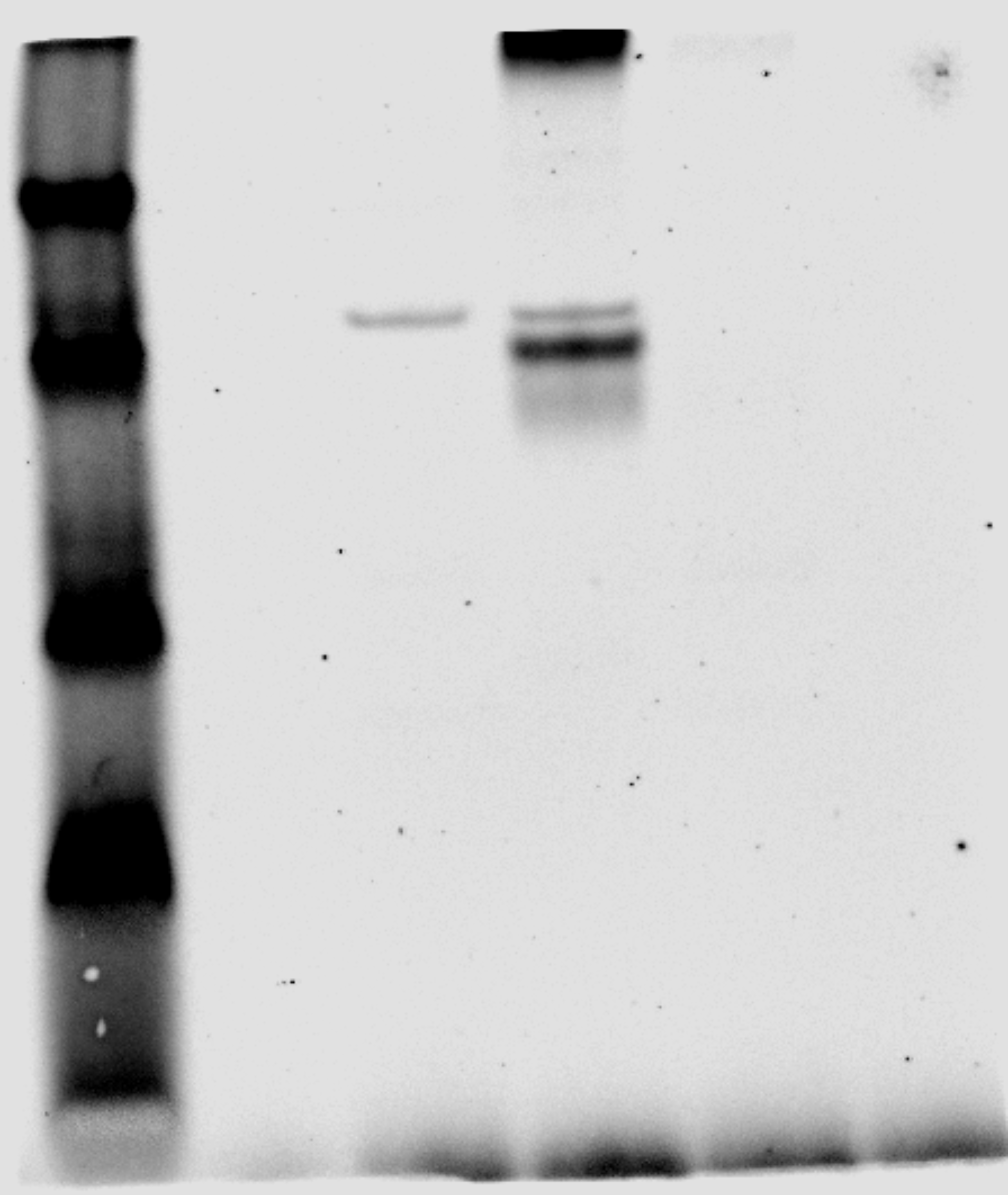

Supplement: Figure 6—figure supplement 1—source data 2. [file elife-89015-fig6-figsupp1-data2.zip › Figure 6-figure supplement 1 raw blots/C - act OT-I RPS3 raw.tif]

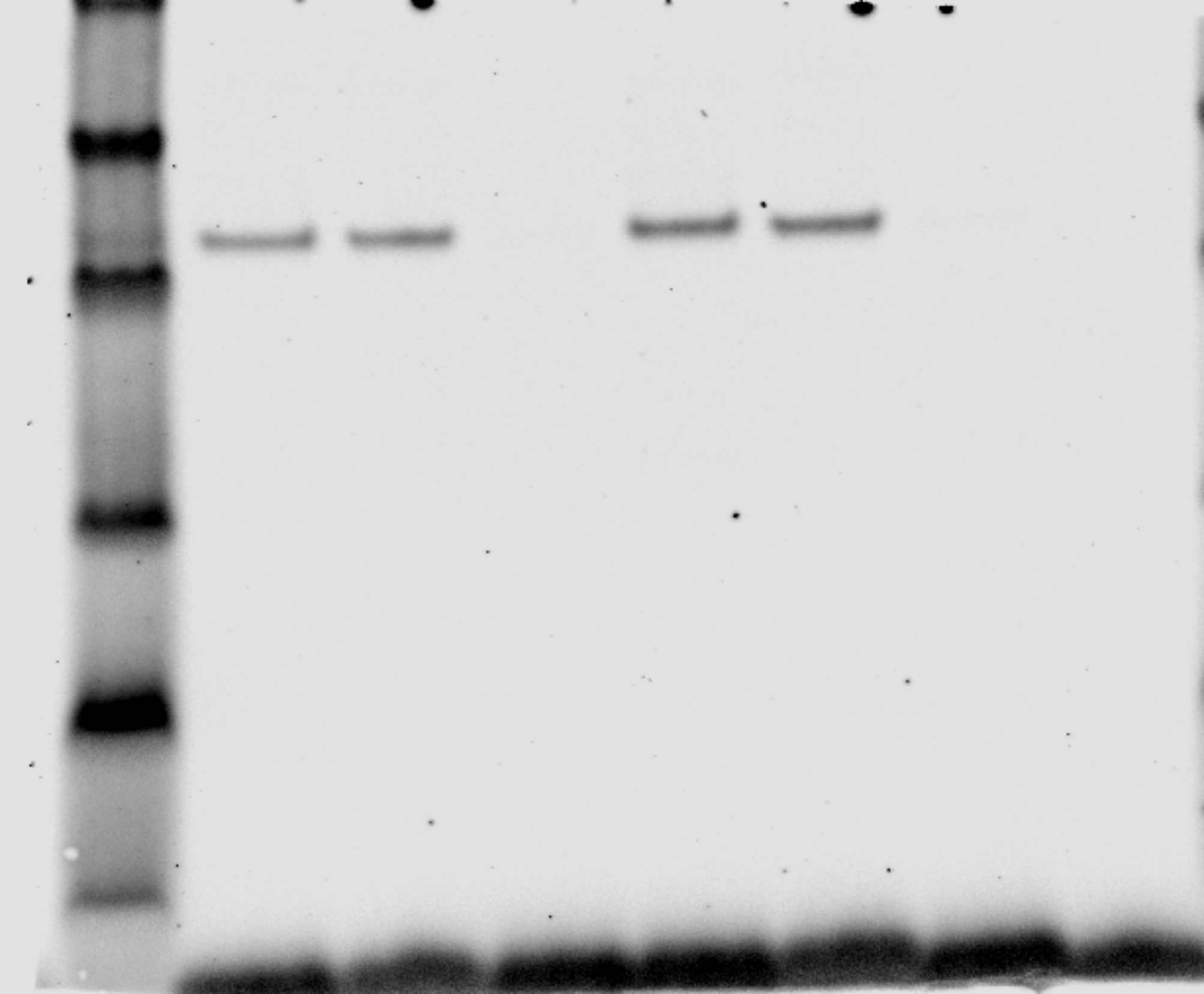

Supplement: Figure 6—figure supplement 1—source data 2. [file elife-89015-fig6-figsupp1-data2.zip › Figure 6-figure supplement 1 raw blots/C - act OT-I RPS6 raw.tif]
